# Understanding morphological variability in a taxonomic context in Chilean diplomystids (Teleostei: Siluriformes), including the description of a new species

**DOI:** 10.7717/peerj.2991

**Published:** 2017-02-16

**Authors:** Gloria Arratia, Claudio Quezada-Romegialli

**Affiliations:** 1 Biodiversity Institute, University of Kansas, Lawrence, KS, United States of America; 2 Departamento de Ciencias Ecológicas, Facultad de Ciencias, Universidad de Chile, Chile; 3 Instituto de Ciencias Naturales Alexander von Humboldt, Facultad de Ciencias del Mar y Recursos Biológicos, Universidad de Antofagasta, Antofagasta, Chile

**Keywords:** Catfishes, Diplomystidae, South America, Freshwaters, Morphology, Variability, Taxonomic diagnoses, Endangered status

## Abstract

Following study of the external morphology and its unmatched variability throughout ontogeny and a re-examination of selected morphological characters based on many specimens of diplomystids from Central and South Chile, we revised and emended previous specific diagnoses and consider *Diplomystes chilensis*, *D. nahuelbutaensis*, *D. camposensis*, and *Olivaichthys viedmensis* (Baker River) to be valid species. Another group, previously identified as *Diplomystes* sp., *D*. spec., *D*. aff. *chilensis*, and *D*. cf. *chilensis* inhabiting rivers between Rapel and Itata Basins is given a new specific name (*Diplomystes incognitus*) and is diagnosed. An identification key to the Chilean species, including the new species, is presented. All specific diagnoses are based on external morphological characters, such as aspects of the skin, neuromast lines, and main lateral line, and position of the anus and urogenital pore, as well as certain osteological characters to facilitate the identification of these species that previously was based on many internal characters. Diplomystids below 150 mm standard length (SL) share a similar external morphology and body proportions that make identification difficult; however, specimens over 150 mm SL can be diagnosed by the position of the urogenital pore and anus, and a combination of external and internal morphological characters. According to current knowledge, diplomystid species have an allopatric distribution with each species apparently endemic to particular basins in continental Chile and one species (*O. viedmensis*) known only from one river in the Chilean Patagonia, but distributed extensively in southern Argentina.

## Introduction

The catfish family Diplomystidae Eigenmann, 1890, endemic to continental waters of the Andean Region (Austral Realm) of South America, represents one of the earliest branching lineages among 43 recent and fossil families of the order Siluriformes (Lundberg & Baskin, 1969; Britz, Kakkassery & Raghaven, 2014). According to morphological studies, Diplomystidae is the sister to all other catfishes (e.g., Lundberg & Baskin (1969) based on the caudal skeleton; Fink & Fink (1981), Fink & Fink (1996), Arratia (1987), Grande (1987) and Pinna (1998) based on miscellaneous morphological characters; Arratia (1992) based especially on characters of the suspensorium), whereas according to molecular evidence, Nematogenyidae Eigenmann, 1927 plus all other loricarioids are the sister of Diplomystidae plus all other catfishes (Sullivan, Lundberg & Hardman, 2006).

**Figure 1:**
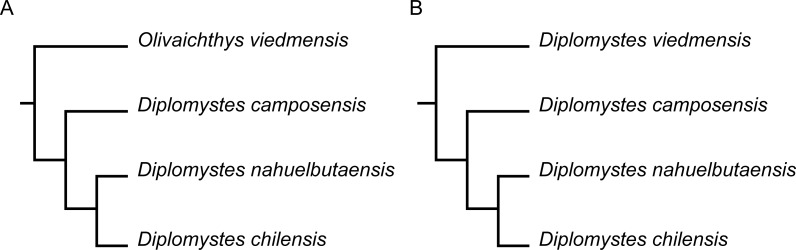
Hypotheses of phylogenetic relationships of species of Diplomystidae. (A) After Arratia (1987), based on 33 morphological characters; (B) after Muñoz-Ramírez et al. (2014) based on molecular evidence. Both topologies are presented to emphasize their congruence.

Different interpretations concerning the taxonomic composition of the family exist. According to the phylogenetic analysis of Arratia (1987; Fig. 1A herein), Diplomystidae includes two genera, *Diplomystes* Duméril, 1856 (with three valid species, *D. chilensis* (Molina, 1782), *D. nahuelbutaensis* Arratia, 1987 and *D. camposensis* Arratia, 1987), and *Olivaichthys* Arratia, 1987 (with *O. viedmensis* Mac Donagh, 1931), which are found in freshwaters of Chile and Argentina, respectively (see also Ferraris, 2003; Ferraris, 2007; López et al., 2008; Cussac et al., 2016). In contrast, Azpelicueta (1994) based on certain morphological characters, interpreted the genus *Olivaichthys* as a synonym of *Diplomystes* and recognized the three subspecies previously described for Argentina as species (*D. cuyanus*, *D. mesembrinus*, and *D. viedmensis*; see also Ferraris, 2003; López et al., 2008). The most recent molecular study, which included Argentinean diplomystids, listed only *D. viedmensis* as a valid species for the country (Muñoz-Ramírez et al., 2014; Fig. 1B herein), confirming the morphological phylogenetic hypothesis of Arratia (1987; Figs. 1A–1B). Considering that in both morphological and molecular hypotheses *Diplomystes* and *Olivaichthys* are monophyletic, we interpret *Olivaichthys* as a valid taxon, an approach that we follow here.

**Figure 2:**
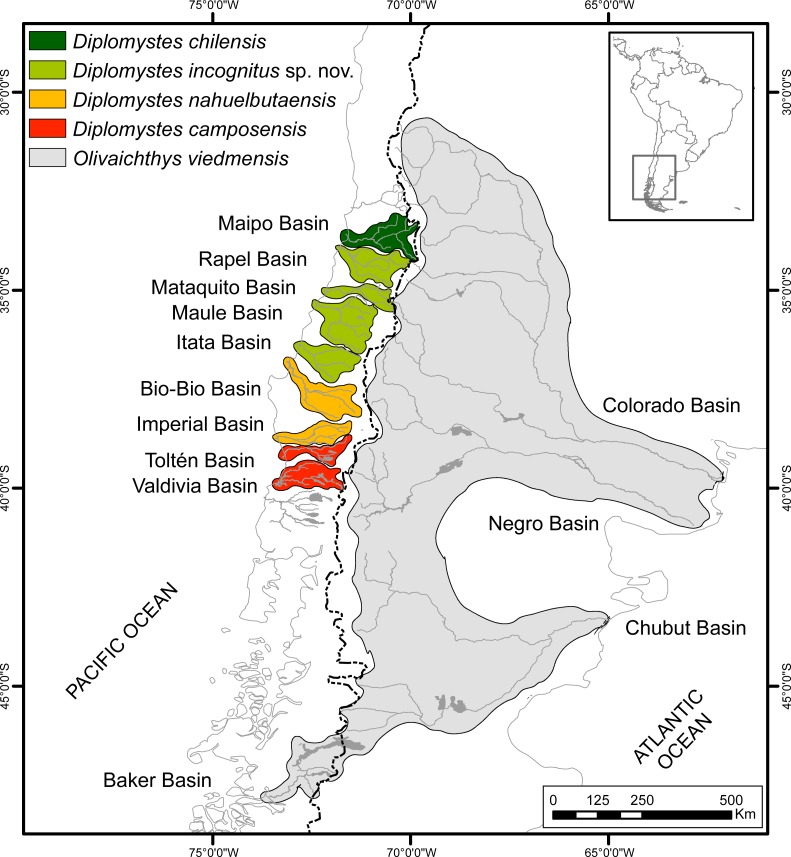
Geographic distribution of diplomystids in Chile and Argentina. Figure based on the specimens studied herein and available literature.

According to current morphological information, four nominal species inhabit the freshwaters of Central and South Chile: *D. chilensis, D. nahuelbutaensis, D. camposensis* (Arratia, 1987; Arratia & Huaquín, 1995; Dyer, 2000; herein), and *Olivaichthys viedmensis*, which is in the Baker River, the southern-most known distribution of diplomystids (Centro de Ecología Aplicada, 2008; Muñoz-Ramírez et al., 2014; present paper). Tentatively, we name this taxon *Olivaichthys viedmensis* until our morphological study in progress that includes specimens from Argentina and Chile is published. Additionally, a group identified as *Diplomystes* spec. from central Chile (Rapel and Maule Basins) was briefly described by Arratia (1987: 63) and Arratia & Huaquín (1995: 44–46, Figs. 20–21) and left without taxonomic assignment until more material would become available. Arratia & Huaquín (1995: 46–50, Figs. 21–24) described the skin of another potential new diplomystid from Copequén River (a northern tributary of Rapel Basin) that was identified as *Diplomystes* aff. *chilensis*, because of its geographic proximity to Maipo Basin, the type locality of *D. chilensis* (Fig. 2). These diplomystids are described here as a new species (see below).

A recent phylogeographic analysis (Muñoz-Ramírez et al., 2014) of Diplomystidae resulted in a topology of the tree identical to the phylogenetic hypothesis based on morphological characters (compare Figs. 1A with 1B) proposed by Arratia (1987: Fig. 38). Few differences between morphological and molecular data and interpretations exist. For instance, Muñoz-Ramírez et al. (2014) placed all species in *Diplomystes* instead of *Diplomystes* and *Olivaichthys*. Other differences concern Chilean populations from Imperial, Toltén and Valdivia Basins (see Fig. 2 for identification of basins) that grouped together so that the authors suggested that *D. nahuelbutaensis* (type locality Imperial Basin) would extend to the south, and if true, then *D. camposensis* would be a synonym of *D. nahuelbutaensis*, and possibly, the population of Toltén Basin is a divergent population or undescribed species. The population of Bío-Bío Basin that appears in their results as genetically distinct would be a new species, not *D. nahuelbutaensis*. Curiously, the authors assumed that the identification of specimens can be determined by their geographic distribution and failed to mention that the diagnoses of the three nominal species, *D. chilensis*, *D. nahuelbutaensis*, and *D. camposensis* are supported by numerous morphological characters (Arratia, 1987: 13, 34, 45) and also by genetic characteristics of *D. nahuelbutaensis* and *D. camposensis* based on specimens from the upper part of Bío-Bío and Imperial Basins, and Toltén and Valdivia Basins, respectively (Campos, Arratia & Cuevas, 1997).

According to Muñoz-Ramírez et al. (2014), individuals inhabiting the rivers between Rapel and Itata Basins comprise a genetic group that they named *Diplomystes* cf. *chilensis*. Again, *D*. cf. *chilensis* from Rapel and Mataquito Basins was mentioned by Muñoz-Ramírez, Victoriano & Habit (2015), with no explanation for the name or of a comparison with *D. chilensis*, nor a comparison with the potential groups identified by Arratia (1987) and Arratia & Huaquín (1995).

All those species appear to have an allopatric distribution and are categorized as in danger of extinction (Arratia, 1987; Campos et al., 1998; Habit, 2005; Habit et al., 2009; MINSEGPRES, 2008), with the exception of *O. viedmensis* that does not yet have an official status since it is a taxon recently discovered in southern Chile (see above). Although there is scarce knowledge of their biology (Arratia, 1983), information about reproduction and general biology of *D. nahuelbutaensis* (Vila, Contreras & Fuentes, 1996; Habit, 2005), general biology of *D. camposensis* (see Cifuentes et al., 2012; Colin, Piedra & Habit, 2012), and the diet of both *Diplomystes* species (Beltrán-Concha et al., 2012) has been reported. Furthermore, information about the karyotypes and chromosomes is available, which are characteristically diagnostic of *D. nahuelbutaensis* and *D. camposensis* (Campos, Arratia & Cuevas, 1997).

All diagnoses of the species of Diplomystidae are heavily based on osteological characters that require study of cleared and stained specimens under microscopes. Thus, any taxonomic assignment of specimens could be incorrect without this morphological analysis. Since the species appear to have an allopatric distribution, information regarding the basin specimens collection is critical to assign a possible identification. However, any taxonomic assignment needs to be confirmed with morphological characters, as done here. Additionally, the skin of diplomystids is highly specialized and diagnostic (Arratia, 1987; Arratia & Huaquín, 1995; herein), but again, the skin requires observation under microscopes (i.e., SEM) that makes its description difficult.

Previous morphological studies (e.g., Arratia, 1987; Arratia, 1992; Arratia & Huaquín, 1995) of Chilean diplomystids were based on relatively a few specimens due to their endangered status. In those studies on diplomystids, Arratia observed marked external body variation that appears to be connected with individual size (age) complicating the identification of particular specimens. She could not study such variability due to the limited sample size availability, which could not be evaluated statistically. During the last 25 years, larger numbers of specimens were collected by non-governmental environmental organizations. The main goals for such collections were to (i) find new localities to establish the overall geographic distribution of Diplomystidae, (ii) clarify the endangered status of the different species, and (iii) collect molecular data from the different populations. Those agencies donated their specimens to us and therefore, our specimens are not the same as those used in the molecular studies by Muñoz-Ramírez et al. (2014) and Muñoz-Ramírez, Victoriano & Habit (2015). These new specimens plus older ones deposited in museums worldwide, are the foundation of this contribution whose main goals are to (1) study and analyze for the first time the little-known ontogenetic variation of body morphology; (2) search for external diagnostic morphological characters to facilitate specific identification; (3) communicate new morphological information that we have compiled; (4) describe a new diplomystid species; and (5) re-evaluate a few diagnostic features characterizing the now five nominal species of the Chilean diplomystids, as well as create an identification key.

### Geographical distribution

According to current information, the Chilean species of Diplomystidae have allopatric distributions (Fig. 2), and seem to be endemic to specific basins, with the possible exception of the widespread *Olivaichthys viedmensis* in the Baker Basin of southern Chile and Argentina.

***Diplomystes chilensis*** (Molina, 1782), with Maipo Basin as the type locality, was recorded in the literature from a few localities in this basin (e.g., Colina and Paine; Leybold, 1859; Philippi, 1866), and most frequently from the “rivers of Santiago,” which refers to area rivers of the Maipo Basin. References to specimens from the rivers of Valparaiso (Aconcagua Basin) have been repeated in the literature following LaCepede (1803: 114); however, no single specimen from this basin in any museum or an illustration from a specimen supposedly collected in Aconcagua Basin has been found. It is doubtful if *D. chilensis* ever lived in the Aconcagua Basin. Consequently, Fig. 2 illustrates the presence of *D. chilensis* only in the Maipo Basin. It is important to note that the last specimens known from this basin are from C Eigenmann, who bought them in the Santiago Central Market in 1919. The survey of Maipo Basin from El Yeso Dam (2,570 m a.s.l.) to Tejas Verdes (9 m a.s.l.) by Duarte et al. (1971) gave negative results. The results of the most recent survey of over 90 sites sampled from 2007 to 2016 between the geographic areas of Aconcagua [32°44′49″S] to the Itata (36°38′34″S) Basins (from coast to subandean regions) by CQ-R also were negative.

***Diplomystes nahuelbutaensis*** Arratia, 1987 has Cautín River, part of the Imperial Basin as the type locality. It is also found in the Bío-Bío Basin (Arratia, 1987; herein). The Loncomilla River belonging to the Maule Basin was also mentioned by Arratia (1987), but after our revisions this has been removed as a locality of *D. nahuelbutaensis*. Other basins (e.g., Itata; Habit, 1994; Muñoz-Ramírez et al., 2014) have been mentioned as inhabited by *D. nahuelbutaensis*. However, we have contrary evidence for the identification of the diplomystids south of Maipo Basin and north of Bío-Bío Basin. See below.

***Diplomystes camposensis*** Arratia, 1987, with Valdivia Basin as the type locality, is the southernmost species of *Diplomystes* (Fig. 2). Later, Colicó Lake (Toltén Basin) was added by Campos, Arratia & Cuevas (1997).

***Diplomystes incognitus*** **sp.** **nov.** Arratia (1987: 65) briefly described specimens identified as *Diplomystes* spec. from Copequén and Tinguiririca Rivers (Rapel Basin) and Maule Basin. In subsequent publications, diplomystids in Rapel Basin were named *Diplomystes* aff. *chilensis* by Arratia & Huaquín (1995). Those living in the Rapel and Mataquito Basins were identified as *Diplomystes* cf. *chilensis* by Muñoz-Ramírez et al. (2014: Table 1). The name *D*. aff. *chilensis* was used by Muñoz-Ramírez et al. because the basins are successively placed south of the Maipo Basin (see Fig. 2). An explanation for *D*. cf. *chilensis* was omitted.

***Olivaichthys viedmensis*** (Mac Donagh, 1931) has a broad distribution in Argentina. It has a restricted distribution in Chile (Fig. 2). This diplomystid has been collected in Baker River, part of Buenos Aires/General Carrera Lake of Argentina and Chile. The Baker River flows west into the Pacific Ocean and is considered one of the longest, most turbulent, and swift-flowing rivers of Chile. The lake, which lies at the boundary between both countries and has a different name in each country, is at low elevation in the Andes Cordillera. In addition to *Olivaichthys*, another catfish (*Hatcheria macraei* (Girard, 1855)) characteristic of the Argentinian Patagonia (Arratia & Menu-Marque, 1981) is found in this locality as well as in Aysén in southern Chile (Zama & Cárdenas, 1984). We assume that the presence of both catfishes in southern Argentina and Chile is natural and not a result of human introduction.

## Material and Methods

### Material studied

Considering the endangered status of *Diplomystes*, museum specimens that were collected long ago are included as well as a significant number of specimens collected from 1989 to 2015 by private environmental organizations. Both and new specimens were collected in the central valley up to the Andean region in Chile, with the exception of one specimen from the Nonguén River (part of the coastal Andalién Basin). The survey of diplomystids in coastal rivers performed by CQ-R during the last nine years—from Aconcagua to Puerto Montt–has given negative results. Most specimens are fixed in ethanol. Some have been cleared and stained (c&s), and one is a dry skeleton (ske). Radiographs were obtained for most specimens. Institutional abbreviations are listed in Sabaj Perez (2014) except for PC, which refers to specimens that are under the care of the first author and will be deposited in the Collections of Fishes of the National Museum of Natural History, Santiago, Chile after completion of the study. All specimens have been kept separated by locality and data collection. Each specimen was measured following standard procedures (outlined below) and provisionally identified following the diagnoses in Arratia (1987) and characterization of cephalic sensory canals, pores, and neuromast lines in Arratia & Huaquín (1995).

**Figure 3:**
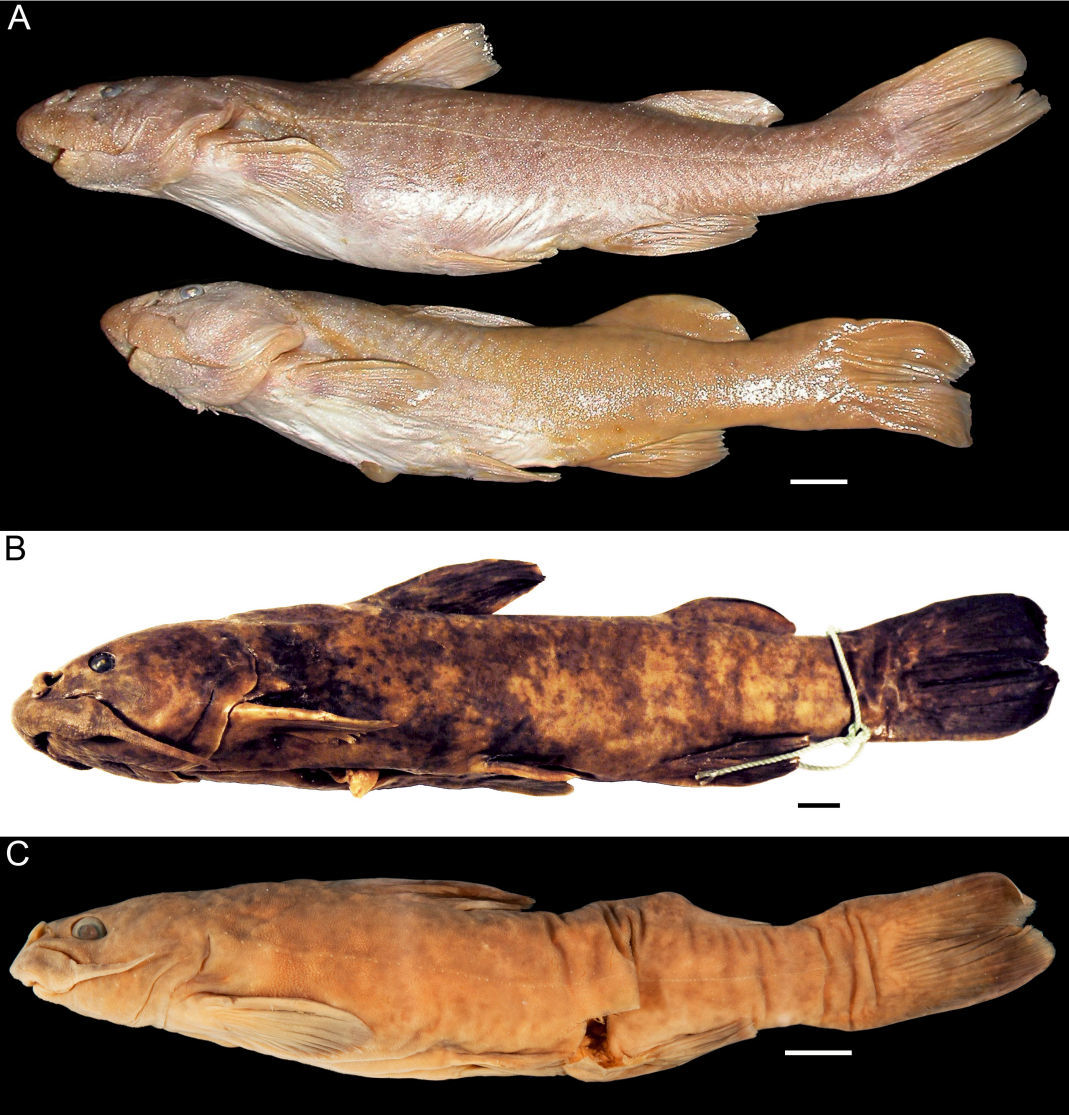
Species of *Diplomystes* in lateral view. (A) *Diplomystes chilensis*, ZMB 6007, 144 and 174 mm SL, Maipo Basin; photograph courtesy of P Bartsch; (B) *Diplomystes nahuelbutaensis*, PC 010391, 211.1 mm SL, Bío-Bío Basin; photograph courtesy of K Sturm; (C) *Diplomystes camposensis* MCZ 54388, 130.5 mm SL; Valdivia Basin; photograph courtesy of MCZ; all copyrights reserved; scale bars = 1 cm.

**Figure 4:**
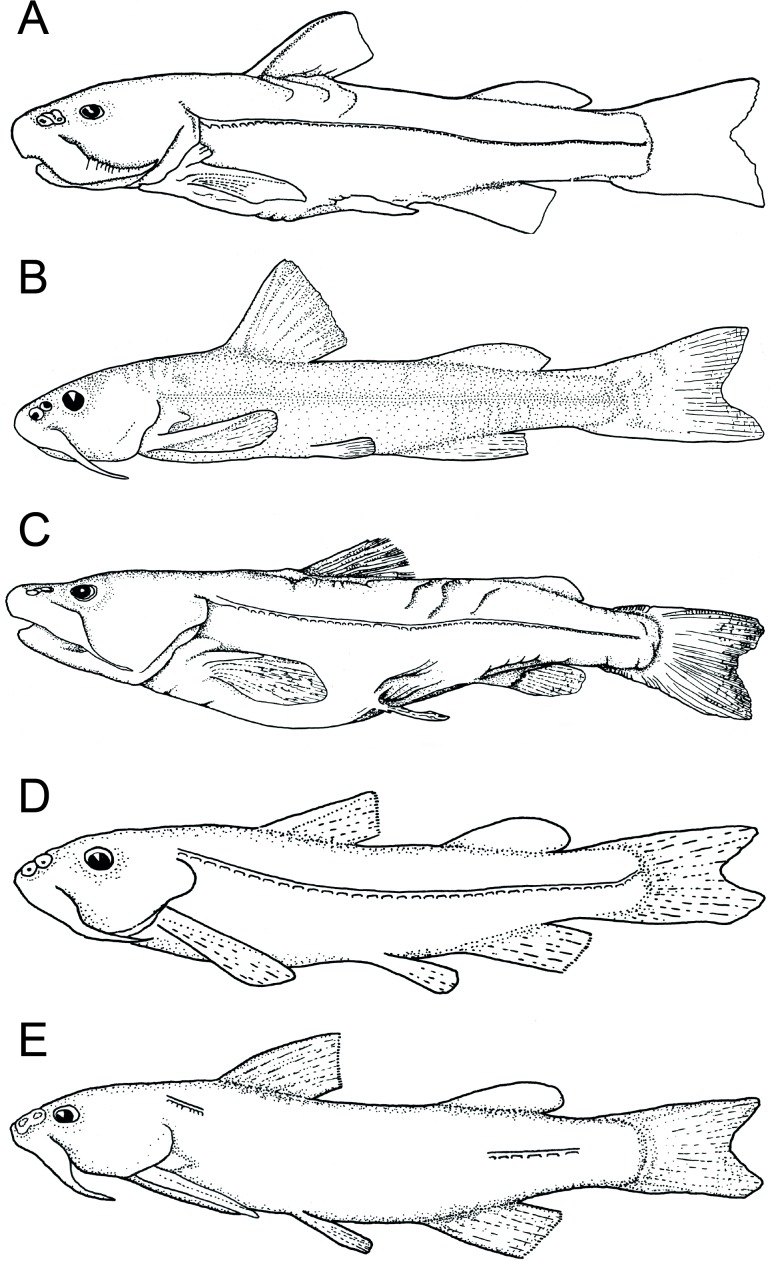
Line drawings of diplomystids in lateral view (A) *Diplomystes chilensis*, MCZ 8290, 153.5 mm SL, Maipo Basin; (B) *Diplomystes incognitus* sp. nov., MNHNCL ICT 7539 (paratype), 108.2 mm SL; Maule Basin; (C) *Diplomystes nahuelbutaensis*, CAS 55423, 222.2 mm SL, Imperial Basin; (D) *Diplomystes camposensis*, KUNHM 19209; (E) *Olivaichthys viedmensis*, PC 01072006, 140.5 mm SL, Baker Basin.

***Diplomystes chilensis*** (Molina, 1782); Figs. 2, 3A, 4A: all from Chile: MNHN B-0584, 2 syntypes of *Arius papillosus* Valenciennes, 184–189 mm SL; [rivers of] Santiago, Región Metropolitana; C Gay, 1832.—MNHN B-0585, 4 syntypes of *Arius papillosus* Valenciennes, 99–148 mm SL (in very poor condition); [rivers of] Santiago; C Gay, 1832.—CAS 13706, 7, 163.0–176.0 mm SL.—CAS 27839 (=IUM 15550), 2, 132.0–147.0 mm SL.—CAS (SU) 23936, 1, 152.3 mm SL; all from Santiago Central Market; C  Eigenmann, 1919.—MCZ 8290, 2, 151.0–153.5 mm SL and 3 disarticulated c&s; rivers of Santiago; Leyboldt-Thayer Expedition, 1865-66.—MCZ 36195, 1, 162.0 mm SL.—ZMB 6007, 2, 144.4–173.9 mm SL; [rivers of] Santiago, Región Metropolitana; R Philippi, 1866?

***Diplomystes nahuelbutaensis*** Arratia, 1987; Figs. 2, 3B, 4C all from Chile: CAS (SU) 55423, holotype, 222.2 mm SL; Cautín River, Lautaro, Región de la Araucanía, 38°31′54″S 72°25′49″W; C Eigenmann, February 13, 1919. —CAS 55424, 6 paratypes, 101.2–141 mm SL; Cautín River, Lautaro, Región de la Araucanía, 38°31′54″S 72°25′49″W; C Eigenmann, February 13, 1919.—NHM 1876-10-2, 1 paratype (ske); no other data.—CAS 55425, 1 c&s partially disarticulated; Cautín River, Lautaro, Región de la Araucanía, 38°31′54″S 72°25′49″W; C Eigenmann, February 13, 1919. —CAS 30875, 1 paratype, 138.0 mm SL; Nonguén River, Concepción, Región del Bío-Bío, 36°49″S 73°03″W; C  Eigenmann, March 20, 1919.—LBUCH 010391, 5, 110.9–185.0 mm SL, 3 c&s, 88.4–146.5 mm SL; Altos del Bío-Bío, Región del Bío-Bío, 37°52′51″S 71°38′59″W; H Thielemann, 1991.—MCZ 61245, 2 paratypes, 104.9–112.3 mm SL; Altos del Bío-Bío, Región del Bío-Bío, 37°40′38″S 72°01′13″W; VH Ruiz & H Oyarzo, March 30, 1984. —ANSP 177913, 4, 12.9–127.1 mm SL; Laja River, 2 km S Tucapel, Concepción, Región del Bío-Bío, 37°40′38″S 72°01′13″W; T Berra & VH Ruiz, November 14, 1992. —ANSP 177914, 2, 31.1–39.8 mm SL; Laja River, La Cantera, 7 km below Salto del Laja, Concepción, Región del Bío-Bío, 37°12′56″S 72° 27′40″W; T Berra & VH Ruiz, December 9, 1992.—MNHNCL P.6668, 151.6 mm SL; Polcura river (near Central Hidroeléctrica Antuco), Bío-Bío Basin, Región del Bío-Bío, 37°17′42″S 71°29′27″W; Peirano-Weisbel & Torres; no other data.—MNHNCL 7007, 3, 122.3–131 mm SL; Piulo River, Bío-Bío Basin, Región del Bío-Bío, 37°42′34″S 71°49′38″W; no other data.—MNHNCL uncat., 135.9 mm SL; Piulo river, near Callaqui, Bío-Bío Basin, Región del Bío-Bío, 37°42′34″S 71°49′38″W; H Thielemann, May, 1996. —MNHNCL uncat., 5, 118.5–175.7 mm SL; Piulo River, Bío-Bío Basin, Región del Bío-Bío, 37°42′34″S 71°49′38″W; no other data.—MNHNCL uncat., 1, 101.9, Bío-Bío River in Calchigue, Región del Bío-Bío, 37°52′33″S 71°39′12″W; no other data.—MNHNCL uncat., 4, 108.4–154.9 mm SL; Queuco River, Bío-Bío Basin, Región del Bío-Bío, 37°49′53″S 71°34′21″W; H Thielemann, January, 8, 1995.—MNHNCL uncat., 3, 96.2–124.2 mm SL; Bío-Bío Basin, Región del Bío-Bío; H  Thielemann, 1994.—MNHNCL uncat., 10, 27.5–136 mm SL; Bío-Bío Basin, Región del Bío-Bío; H Thielemann, March, 1998.—MNHNCL uncat., 4, 104.4–127.1 mm SL; Altos del Bío-Bío, Región del Bío-Bío, 37°52′51″S 71°38′59″W; H Thielemann, 1996; [PC 010396].—MNHNCL uncat., 5, 133.2–170.0 mm SL; Bío-Bío River; August 1997; no other data; [PC 080097].

***Diplomystes camposensis*** Arratia, 1987; Figs. 2, 3C, 4D; all from Chile: CAS 55428, 2, 73.7–78.7 mm SL; Riñihue Lake, Valdivia Basin, Región de los Ríos, 39°48′16″S 72°23′13″W; C Eigenmann, March 16, 1919.—IZUA 2807, 2 paratypes, 106.7–169.6 mm SL; Riñihue Lake, Valdivia Basin, Región de los Ríos, 39°48′16″S 72°23′13″W; R  Arriagada; November 4, 1975. —IZUA 3303b, 2 paratypes, 130.2–130.5 mm SL; Riñihue Lake, Valdivia Basin, Región de los Ríos, 39°48′16″S 72°23′13″W; R Arriagada; November 4, 1975. –IZUA 4086, 1, 100.0 mm SL; Leufucade River, Lanco, Valdivia Basin, Región de los Ríos, 39°27′35″S 72°46′21″W; R Arriagada, March 31, 1986. —IZUA 4412, 1, 151.1 mm SL; Colicó Lake, Toltén Basin, Región de la Araucanía, 39° 04′37″S 71°59′52″W; R Arriagada, November 29, 1989. —IZUA 4413, 1, 199.7 mm SL; Colicó Lake, Toltén Basin, Región de la Araucanía, 39°04′37″S 71° 59′52″W; R Arriagada, November 29, 1989. —IZUA 4500, 6 c&s, 101.1–145.5 mm SL and 10, 78–165.0 mm SL; San Pedro River at Los Lagos, Región de los Ríos, 39°51′23″S 72°47′39″W; I Ojeda, January 22, 1989 [= previously identified as PC 220189]. —IZUA uncat, 7, 80.1–130.5 mm SL; Riñihue Lake, Valdivia Basin, Región de los Ríos, 39°48′16″S 72°23′13″W; no other data. —KUNHM 19210, 1 paratype (disarticulated c&s); San Pedro River, Purey, Valdivia Basin, Región de los Ríos, 39°49′06″S 72°51′50″W; G  Arratia & H Díaz, February 19, 1977. —LBUCH 110276, 2 paratypes c&s, 100.8–147.6 mm SL; PC 120276, 1 paratype (disarticulated c&s); San Pedro River, Los Lagos, Valdivia Basin, Región de los Ríos, 39°51′23″S 72°47′39″W; G Arratia, February, 1976. —LBUCH 110276, 10, 78–165.0 mm SL; San Pedro River at Los Lagos, Valdivia Basin, Región de los Ríos, 39°51′23″S 72°47′39″W; I Ojeda, February, 1976. —LBUCH 09102007, 4, 130.7–192.4 mm SL; Riñihue Lake, Valdivia Basin, Región de los Ríos, 39°48′16″S 72°23′13″W; coll. unknown, October 9, 2007. –LBUCH 10102007, 1, 183.4 mm SL; Riñihue Lake, Valdivia Basin, Región de los Ríos, 39°48′16″S 72°23′13″W; coll. unknown, October 10, 2007. —LBUCH 10102007, 2, 141.4–151.8 mm SL; Riñihue Lake at Desaguadero, Valdivia Basin, Región de los Ríos, 39°46′32″S72°27′23″W; coll. unknown, October 10, 2007. —MCZ 54388, 1 paratype, 130.51 mm SL; San Pedro River, Los Lagos, Valdivia Basin, 39°51′23″S 72°47′39″W; G Arratia, March 22, 1979.

**Remarks**. The holotype (IZUA 3302) was lost in the fire of December 3, 2007 which destroyed the Institute of Zoology of the Austral University, Valdivia.

***Diplomystes*** ***incognitus*** **sp. nov.**; Figs. 2 and 4B: all from Chile. See list of material under the description of the new species.

***Olivaichthys viedmensis*** (Mac Donagh, 1931); Figs. 2 and 4E: all from Chile: LBUCH 01072006, 2, 126.8–140.5 mm SL, plus seven larvae with only the anterior part of the body. Baker River, Región de Aysén del General Carlos Ibáñez del Campo, 47°12′10″S 72°37′51″W; August 15, 2006; no other data.

### Methods

Specimens cleared and stained for both bone and cartilage were prepared following the technique of Dingerkus & Uhler (1977) with modifications outlined in Arratia & Schultze (1992).

**Figure 5:**
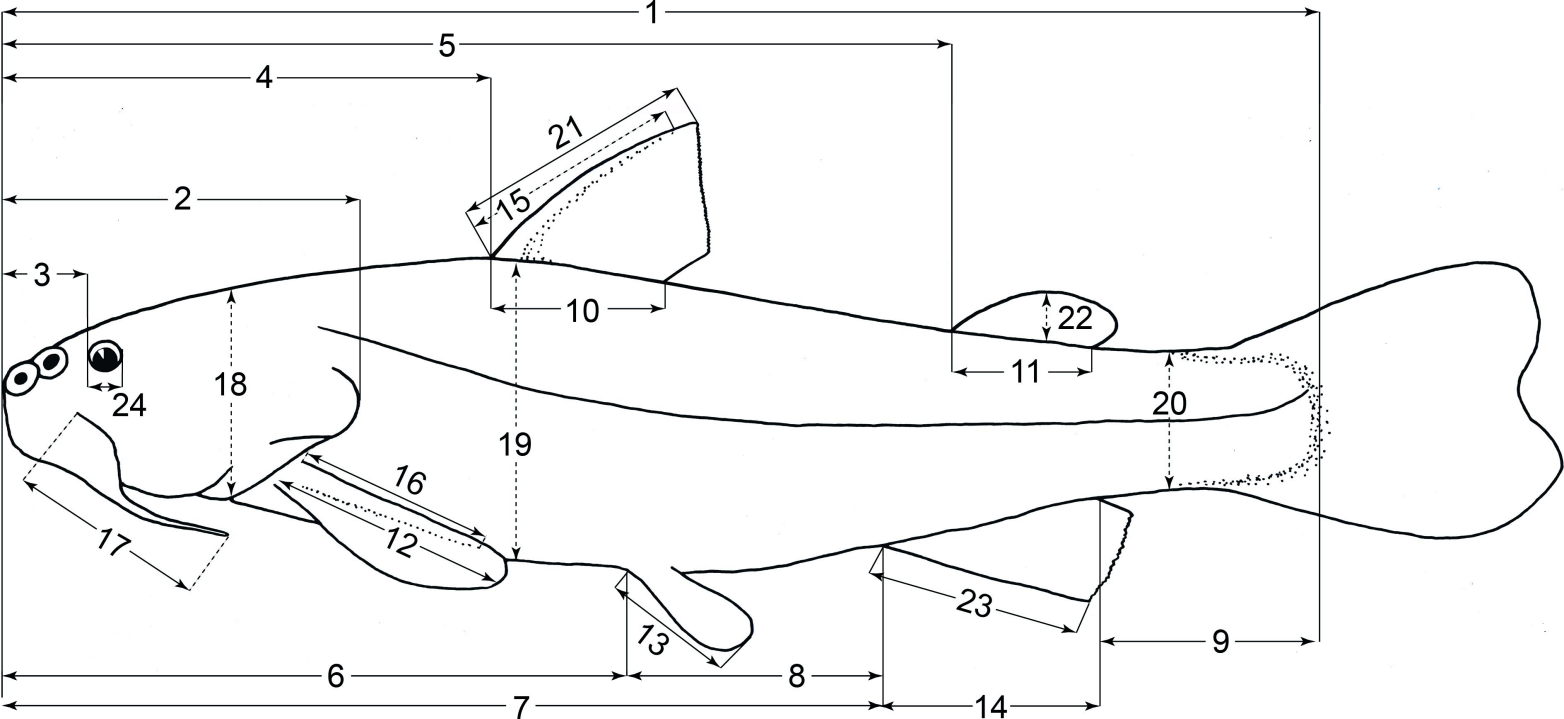
Morphometric character terminology used herein, in specimens in lateral view. 1, standard length; 2, head length; 3, preorbital length; 4, predorsal-fin length; 5, preadipose-fin length; 6, prepelvic-fin length; 7, preanal-fin length; 8, distance between pelvic and anal fin insertions; 9, peduncle length; 10, dorsal-fin base length; 11, adipose-fin base length; 12, pectoral-fin length; 13, pelvic-fin length; 14, anal-fin base length; 15, dorsal-spine length (bony part); 16, pectoral-spine length (bony part); 17, barbel length; 18, head depth; 19, maximum body depth; 20, peduncle depth; 21, dorsal-fin depth; 22, adipose-fin depth; 23, anal-fin depth; 24, orbital diameter.

Morphometric character terminology used herein, is illustrated in Fig. 5, with the following exceptions: interorbital width (distance between dorsal border of orbits), head width (taken at the level of opercle); body width (taken below dorsal fin origin, at mid flank), and peduncle width (taken at the level where peduncle depth was measured). Body measurements were obtained from all studied specimens on the left side of each specimen with a digital caliper reading to 0.1 mm. Different body ratios were calculated and expressed in percent of SL and HL.

Using external morphometric characters, we addressed two issues. First, as changes throughout ontogeny in these species have been observed (G Arratia, pers. obs., 1992, 2000; see results), we were interested in describing and evaluating statistical differences in all morphometric characters among *Diplomystes* species. We focused our analysis between *D. nahuelbutaensis* and *D. camposensis* to achieve proper statistical validity. Due to the limited museum sample size *D. chilensis* and *Diplomystes incognitus* sp. nov. were excluded. We used an analysis of covariance (ANCOVA) with SL as the covariate, to evaluate whether the population means of each morphometric character were statistically different between species, while controlling for the effects of SL. In other words, we calculated linear regressions for each morphometric character per species separately, and then with the ANCOVA, to test for differences in slopes and intercepts between regression lines of both species. In this analysis, if difference in slopes are significant, this implies that the rate of growth for a trait is different between species. If the intercepts differ (but not the slopes), it indicates that the mean of the trait differs consistently through ontogeny but there are no differences in the growth rate. To compare individuals of the same size range, smaller (SL < 90 mm) and larger (SL > 180 mm) individuals were eliminated from this analysis. Normality was checked with q–q plots.

Secondly we evaluated whether morphometric characters can discriminate among the three species of *Diplomystes* and *Diplomystes incognitus* sp. nov., excluding younger specimens (SL < 90 mm). For this, a linear discriminant analysis (LDA) was used. This standard statistical method provides a linear combination of morphological variables that allows the most efficient discrimination among groups, e.g., species (Legendre & Legendre, 2012). To maximize the number of individuals with complete measurements, the following characters were selected for the LDA: (1) head length, head width, head depth, predorsal-fin length, prepelvic-fin length, preanal-fin length, pectoral-fin length, pelvic-fin length, and caudal-fin length in percentage of SL; and (2) barbel length, preorbital length, mouth width, interorbital width and eye diameter in percentage of HL. The LDA was performed in R v 3.1.2 (R Core Team, 2016) with the MASS package (Venables & Ripley, 2002), reporting the jackknifed posterior classification of species (CV = TRUE) (Borcard, Gillet & Legendre, 2011). Graphical operations were performed with the ade4 package (Dray & Dufour, 2007) with default options.

The electronic version of this article in Portable Document Format (PDF) will represent a published work according to the International Commission on Zoological Nomenclature (ICZN), and hence the new names contained in the electronic version are effectively  published under that Code from the electronic edition alone. This published work and the nomenclatural acts it contains have been registered in ZooBank, the online registration system for the ICZN. The ZooBank LSIDs (Life Science Identifiers) can be resolved and the associated information viewed through any standard web browser by appending the LSID to the prefix http://zoobank.org/. The LSID for this publication is: urn:lsid:zoobank.org:pub:D22D3881-ED19-470C-8BAF-0CA306EA073B. The online version of this work is archived and available from the following digital repositories: PeerJ, PubMed Central and CLOCKSS.

## Results

### Morphometric analyses

Considering individuals 90 mm SL and greater: summary statistics of morphometric measurements conducted on 14 specimens of *Diplomystes chilensis* (132–191 mm SL), 10 specimens of *Diplomystes incognitus* sp. nov. (98.3–179.5 mm SL), 50 specimens of *D. nahuelbutaensis* (96.9–222.2 mm SL), and 59 specimens of *D. camposensis* (94.9–202.5 mm SL) are shown in Table 1. Although some mean values differ between species, there is an overlap in most ratios and as such, they are not diagnostic characters. To avoid misinterpretations of means and ranges with reduced number of specimens, changes along the ontogeny are described only between *D. nahuelbutaensis* and *D. camposensis.*

**Table 1:** Morphometric data of *Diplomystes* species.

	*D. chilensis* (*n* = 14)			*D. incognitus* sp. nov. (*n* = 10)			*D. nahuelbutaensis*(*n* = 50)			*D. camposensis* (*n* = 59)		
	Mean	SD	Range	Mean	SD	Range	Mean	SD	Range	Mean	SD	Range
Standard length	150.6	16.4	132.0–191.0	129.9	26.3	98.3–179.5	131.6	27.1	96.9–222.2	136	23.6	94.9–202.5
**Percentage of standard length**												
Head length	25	1.1	23.5–26.9	24.5	2.3	20.2–26.7	27.3	1.5	24.1–30.4	29.3	1.8	23.3–32.3
Predorsal-fin length	34.7	1.7	31.4–37.9	34.3	2.3	30.7–38.3	38.3	2.5	30.4–44.2	39.3	2.7	26.9–45.9
Preadipose-fin length	66	2.1	63.3–69.7	64.8	1.9	61.2–66.7	68.4	3.8	54.3–76.0	70.5	3.3	61.3–76.4
Prepelvic-fin length	46.7	2.3	40.5–50.4	46.4	2	43.8–49.5	49.9	2.2	43.9–54.6	52	2.5	45.5–57.5
Preanal-fin length	64.2	1.4	62.8–67.1	62.2	1.6	60.0–64.9	65	2.5	56.1–69.8	65.8	2.1	60.5–71.0
Dorsal-fin length	21.6	1.5	19.8–24.0	21.2	2	18.5–23.7	21.8	2.4	16.8–27.0	21.4	1.6	16.8–26.1
Adipose-fin length	21.6	1.2	20.1–22.9	22.4	2	19.9–25.1	20.5	2.6	15.6–26.9	19.4	1.7	15.8–22.6
Pectoral-fin length	19.9	0.7	19.0–21.2	19.3	2.2	13.9–21.6	20.7	1.7	16.7–25.3	19.9	1.6	17.1–26.3
Pelvic-fin length	13.2	0.8	11.3–14.4	14.2	1.6	12.5–17.7	12.8	1.1	10.6–15.2	13	1.3	10.1–16.2
Anal-fin length	20.7	1	19.2–21.6	21.5	1.6	20.3–24.9	20.4	1.6	16.2–24.1	19.8	1.4	14.6–22.9
Caudal-fin length	21	1.7	17.6–23.5	18.4	3	13.2–24.3	20	2.7	15.8–30.3	19.3	3.3	6.0–25.0
Pectoral spine length	14.5	2.9	11.6–19.3	18.5	3.3	15.4–25.5	17	2.7	11.8–22.0	19	2.3	9.9–23.1
Dorsal spine length	16.8	1.4	14.8–18.3	17.9	2.4	12.9–21.9	16.8	2.2	11.7–20.7	16.2	1.9	12.3–20.4
Caudal peduncle length	20.1	3.3	16.8–24.7	24.8	0.6	24.3–25.6	22.1	3.1	18.4–27.2	22	0.9	20.5–23.8
Body depth	21	1.9	18.0–23.1	19.1	2.4	15.5–24.3	22.8	2.3	19.0–27.7	24	2.9	17.6–30.9
Head depth	13	0.8	11.3–14.2	15	1.4	13.7–18.6	16.3	2.2	11.9–20.2	17.4	1.6	13.8–21.6
Caudal peduncle depth	10.1	0.4	9.4–10.6	10.3	1.5	8.6–14.0	11	1	8.9–13.7	11.5	1.2	9.1–15.2
Dorsal-fin depth	17	1.1	15.5–18.7	19.5	2.5	17.1–25.1	18.1	1.6	14.7–21.3	17.2	1.5	13.9–21.4
Adipose-fin depth	5.6	1	3.8–7.0	5	1.2	3.9–7.3	4.7	1.1	2.9–7.6	5.7	0.8	3.7–8.3
Anal-fin depth	15	4.4	5.1–17.3	17.7	2	15.3–21.7	15.5	1.4	12.4–18.0	15.8	2	11.6–20.8
Head width	18.3	1.8	16.0–21.5	19.6	1.8	16.0–22.0	21.3	1.6	17.2–24.5	22.7	1.5	18.1–26.2
Body width	17.2	2.4	15.5–19.0	17.3	1.6	14.8–20.1	21.2	1.6	17.9–24.6	22.2	1.4	19.7–25.4
Caudal peduncle width	4.4	0.3	4.2–4.6	4.6	1.5	2.3–7.0	4	0.9	2.5–6.7	5.3	0.9	3.4–7.1
**Percentage of head length**												
Head depth	52	2.6	48–55	62	7.3	51–72	60	8	46–77	59	5.5	49–79
Head width	73	7.8	62–85	82	10.6	60–98	78	5.4	60–89	78	5.7	63–96
Barbel length	51	10	39–77	64	9.6	49–80	60	10.2	32–77	56	5.8	40–66
Preorbital length	34	3	29–39	37	4.4	30–43	35	5.5	25–46	36	2.6	31–42
Mouth width	45	6.6	36–57	42	7	30–51	45	4.5	31–59	43	6.2	31–60
Inter–orbital width	32	4	26–40	34	5.9	26–44	36	4.2	27–44	34	3.8	26–44
Eye diameter	12	0.9	11–14	13	2.4	10–17	12	2.5	8–19	14	1.9	9–19
**Other measures**												
Distance between dorsal-fin origin and adipose-fin origin in % of SL	31	2.1	28–34	31	2.3	25–34	30	3.7	23–43	31	3.6	23–46
Distance between pelvic-fin origin and anal-fin origin (DPvA) in % of SL	18	2.9	15–27	16	1.5	14–19	15	1.6	12–19	14	1.7	11–18
Pelvic-fin length/DPvA	76	9.5	52–88	90	7.3	76–100	86	12	62–112	95	12.8	56–134
Dorsal-fin depth/Dorsal-fin length	80	9.3	67–89	92	11.9	77–112	84	7.4	73–110	81	6.8	64–96
Dorsal-spine length/Dorsal-fin length	78	10.3	64–93	85	12.5	70–112	78	9	56–97	76	8.7	58–92
Dorsal-spine length/Dorsal-fin depth	99	8.2	86–110	92	10.2	72–106	93	8.8	66–114	94	8.8	77–115
Adipose-fin depth/adipose-fin length	26	4.6	17–32	22	4.2	16–29	23	4.4	16–40	29	4.3	21–39
Eye diameter/Preorbital length	38	6.1	29–52	39	11	25–63	35	9.5	22–63	43	7.9	27–62

**Figure 6:**
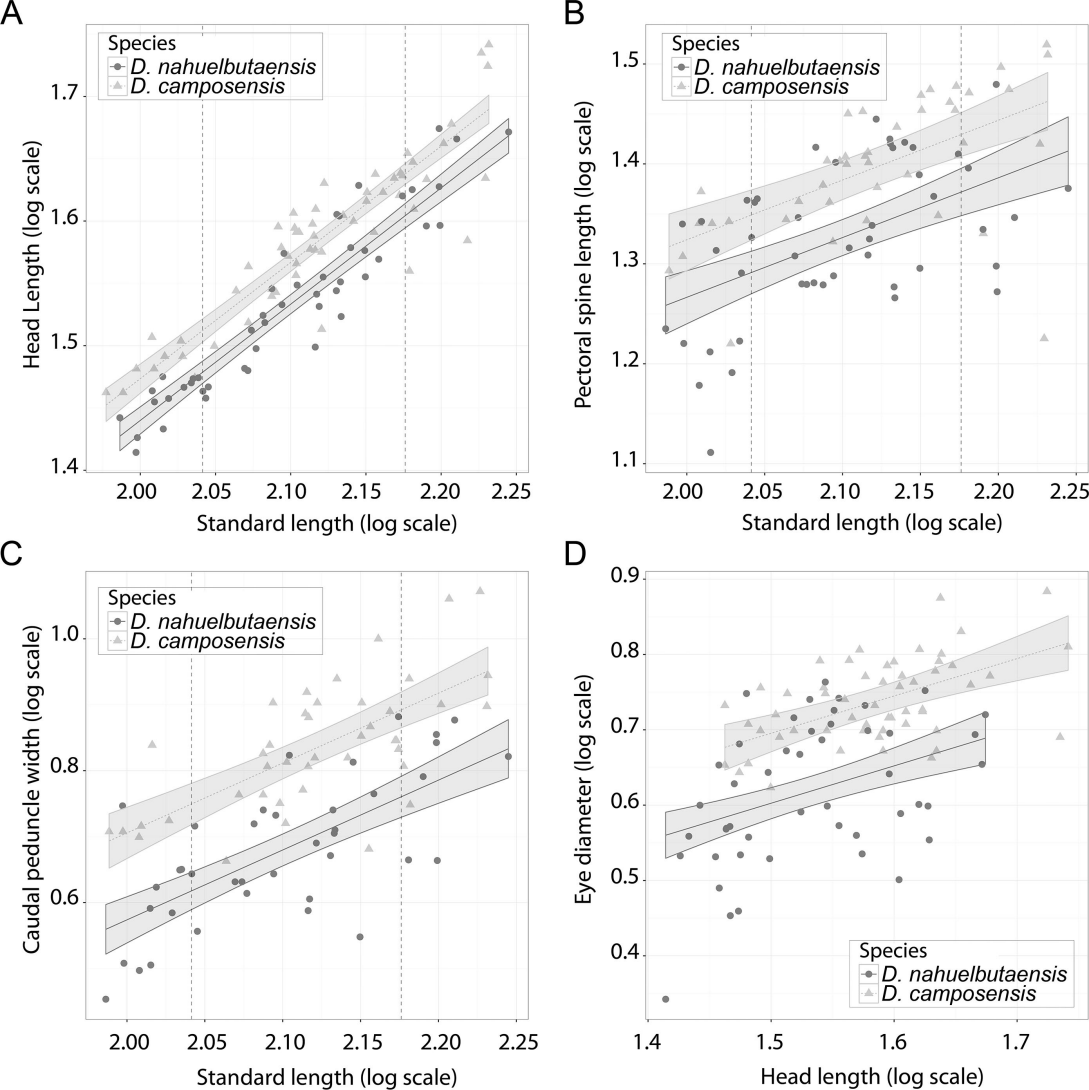
Scatterplots of selected morphometric characters that exhibit growth differences between *Diplomystes nahuelbutaensis* and *D. camposensis*. Each plot includes the linear regression and 95% confidence interval calculated for each species. (A) head length relative to SL; (B) pectoral-spine length relative to SL; (C) caudal peduncle width relative to SL; (D) eye-diameter relative to head length. For the (A)–(C), the dotted vertical lines indicate 110 mm in SL (2.041 in log_10_ scale) and 150 mm in SL (2.176 in log_10_ scale).

Comparisons between *D. nahuelbutaensis* and *D. camposensis* suggest that for a given length *D. nahuelbutaensis* has shorter head and pectoral spine length, a narrower caudal peduncle, and smaller eye diameter (Figs. 6A–6D). In these cases, the slope of the regression lines is equal (ANCOVA *F*_1,99_ = 1.29, *p* > 0.25 for head length; ANCOVA *F*_1,85_ = 0.009, *p* > 0.92 for pectoral spine length; ANCOVA *F*_1,77_ = 0.13, *p* > 0.71 for caudal peduncle width; and ANCOVA *F*_1,99_ = 0.64, *p* > 0.42 for eye diameter), but the intercept is different (ANCOVA *F*_1,100_ = 40.22, *p* < 0.001 for head length; ANCOVA *F*_1,86_ = 18.45, *p* < 0.001 for pectoral spine length; ANCOVA *F*_1,78_ = 59.54, *p* < 0.001 for caudal peduncle width; and ANCOVA *F*_1,100_ =41.52, *p* < 0.001 for eye diameter). These results show that although the rate of growth of these characters is similar across the species examined here, there are consistent differences throughout ontogeny for these morphological features between species.

**Figure 7:**
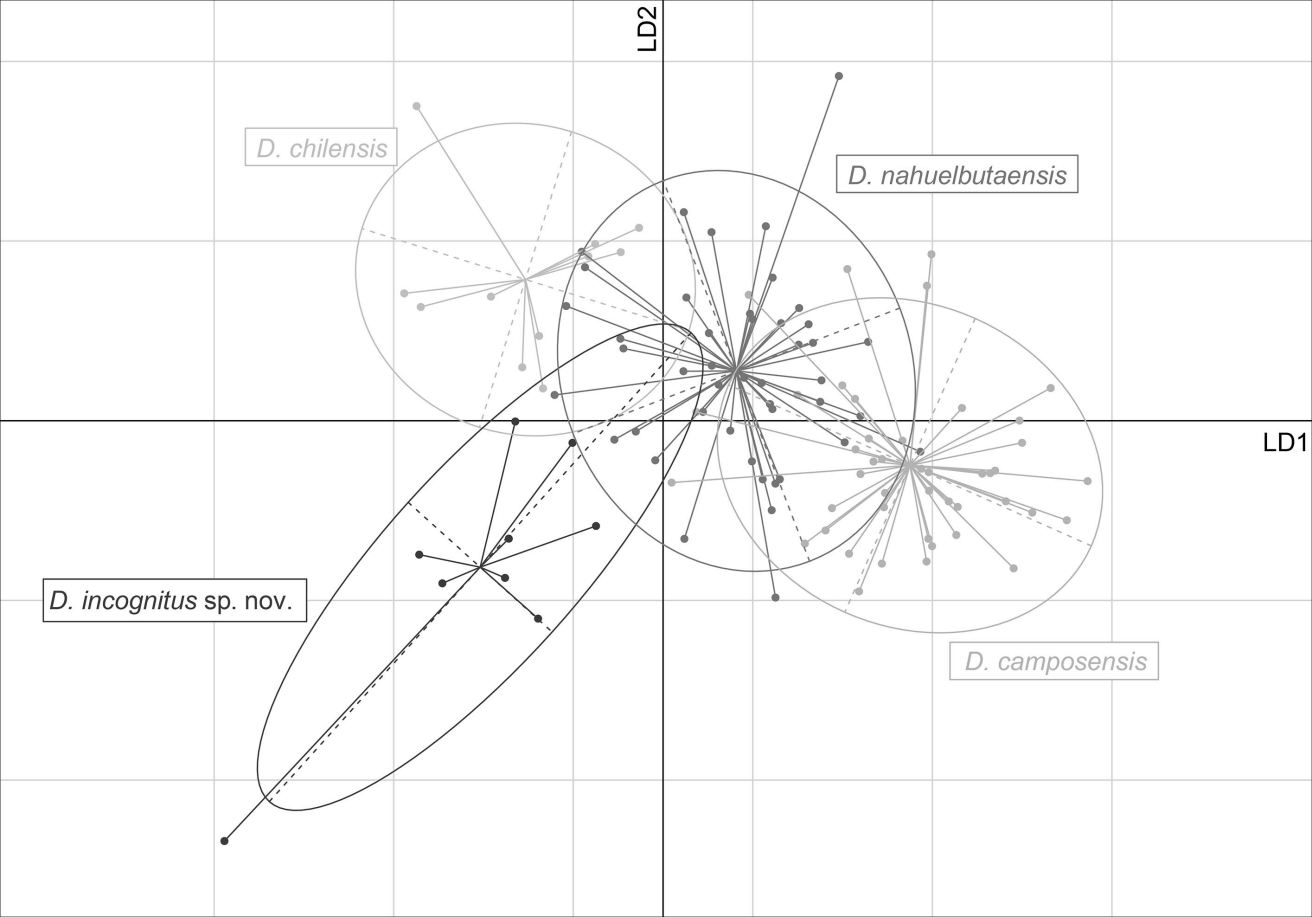
Scatterplot of canonical scores of linear discriminant analysis for selected morphometric characters for *Diplomystes chilensis*, *D. incognitus* sp. nov., *D. nahuelbutaensis* and *D. camposensis*. Each group of points includes the default interval of confidence and the centroid calculated with ade4 Dray & Dufour, 2007. Abb.: LD1, linear discriminant 1; LD2, linear discriminant 2.

The first linear discriminant (LD) explained 67.9% of the variance, whereas the second LD explained 26.2% of the variance, showing that the LDA displays good discriminatory power among species (Fig. 7). The coefficients of the linear discriminants are shown in Table 2: variables with higher linear discriminant coefficients are head length, pelvic-fin length and eye diameter on the LD1; whereas pelvic-fin length, head width and preanal-fin length score highest on LD2. Overall, posterior species classification is highly accurate —88 of 109 individuals were correctly classified (81%; Table 3).

**Table 2:** Coefficients of linear discriminant analysis (LDA) for morphometric data of *Diplomystes* species.

	LD1	LD2	LD3
**Measures in percent of standard length**			
Caudal fin length	0.024	0.086	0.021
Head length	0.396	0.073	0.228
Predorsal-fin length	0.110	0.068	−0.133
Prepelvic-fin length	0.140	−0.107	0.063
Preanal-fin length	−0.046	0.341	−0.083
Pectoral-fin length	0.023	0.249	−0.374
Pelvic-fin length	−0.134	−0.464	0.169
Head depth	0.043	−0.132	−0.073
Head width	0.185	−0.287	−0.020
**Measures in percent of head length**			
Barbel length	−0.013	−0.033	−0.085
Preorbital length	0.009	−0.033	0.173
Mouth width	0.003	0.035	−0.005
Inter-orbital width	0.002	0.046	−0.043
Eye diameter	0.244	−0.150	0.076

**Table 3:** Classification of individuals over 90 mm in SL with the linear discriminant analysis method.

Prior classification with morphology		Jackknifed posterior classification with LDA				
Species	*n*	*D. chilensis*	*D. incognitus* sp. nov	*D. nahuelbutaensis*	*D. camposensis*	% Correctly classified
*D. chilensis*	11	9	0	2	0	82%
*D. incognitus* sp. nov	9	1	6	2	0	67%
*D. nahuelbutaensis*	45	5	1	34	5	76%
*D. camposensis*	44	0	1	4	39	89%

In summary, although the ranges in most characters show overlap between species, a finer analysis reveals significant differences in morphological traits among *Diplomystes* species when analyzed through ontogeny. This implies that most external measures are not diagnostic but change for most species in the same way, maintaining differences through ontogeny. When combined in a standard multivariate analysis, our results show that the LDA permits the classification of individuals of three nominal species of *Diplomystes* and the newly described *D. incognitus* sp. nov. using a set of external morphometric characters.

### Body shape, fins, and ontogenetic changes

Some body proportions of the three species of *Diplomystes* become markedly different throughout ontogeny, especially with regard to the size and position of the fins (see above) and the position of the anus and urogenital pore, which are positioned closely in diplomystids (Arratia, 1987).

**Figure 8:**
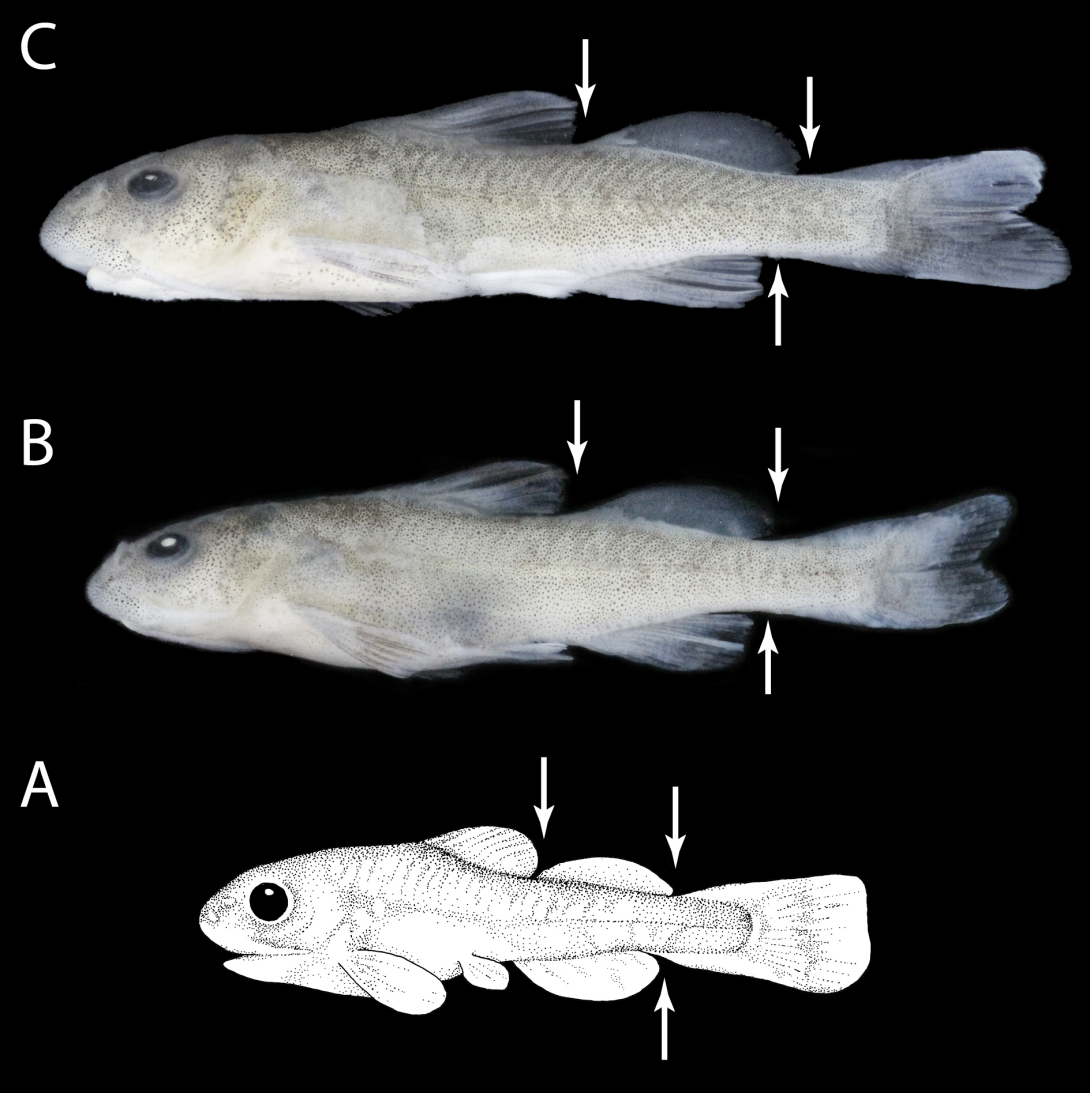
Three freshly collected and preserved specimens of *Diplomystes nahuelbutaensis* and *Diplomystes incognitus* sp. nov. (A) *D. nahuelbutaensis*, ANSP 177914, 30.7 mm SL; (B) *D. incognitus* sp. nov. MNHNCL ICT 7541 39.5 mm SL and (C) *D. incognitus* MNHNCL ICT 7541 41 mm SL. Arrows point to position of the median fins to highlight their relative change through ontogeny. (A) Drawn by G Arratia.

All fins are large and separated by a short distance; it appears as if the fins are continuous in small specimens (under 40 mm SL) of *D. nahuelbutaensis*, *Diplomystes incognitus* sp. nov., and *D. camposensis*. Note, for instance, the large size and almost rounded shape of the paired and unpaired (dorsal, adipose, and anal) fins of *Diplomystes nahuelbutaensis* illustrated in Fig. 8A (see also Lundberg, Berra & Friel, 2004). The pectoral fins are so large that they almost overlap with the pelvic fins. The pelvic fins reach the origin of the anal fins and may extend further caudad. The posterior margin of the adipose and anal fins almost reach the expanded anterior margin of the caudal fin, which, in the smallest specimens, has an almost straight posterior margin (Fig. 8A). As a consequence of the large size of the pelvic fins, the anus and urogenital pore lie between the two pelvic fins in the smallest specimens. As similar features are observed in small specimens of *D. nahuelbutaensis*, *D. camposensis*, *Diplomystes incognitus* sp. nov. (Figs. 8B–8C), and *Olivaichthys viedmensis*, we hypothesize that *D. chilensis* has a similar early development of the fins. There is one available specimen under 133 mm SL (CAS 27839, 132.0 mm SL).

**Figure 9:**
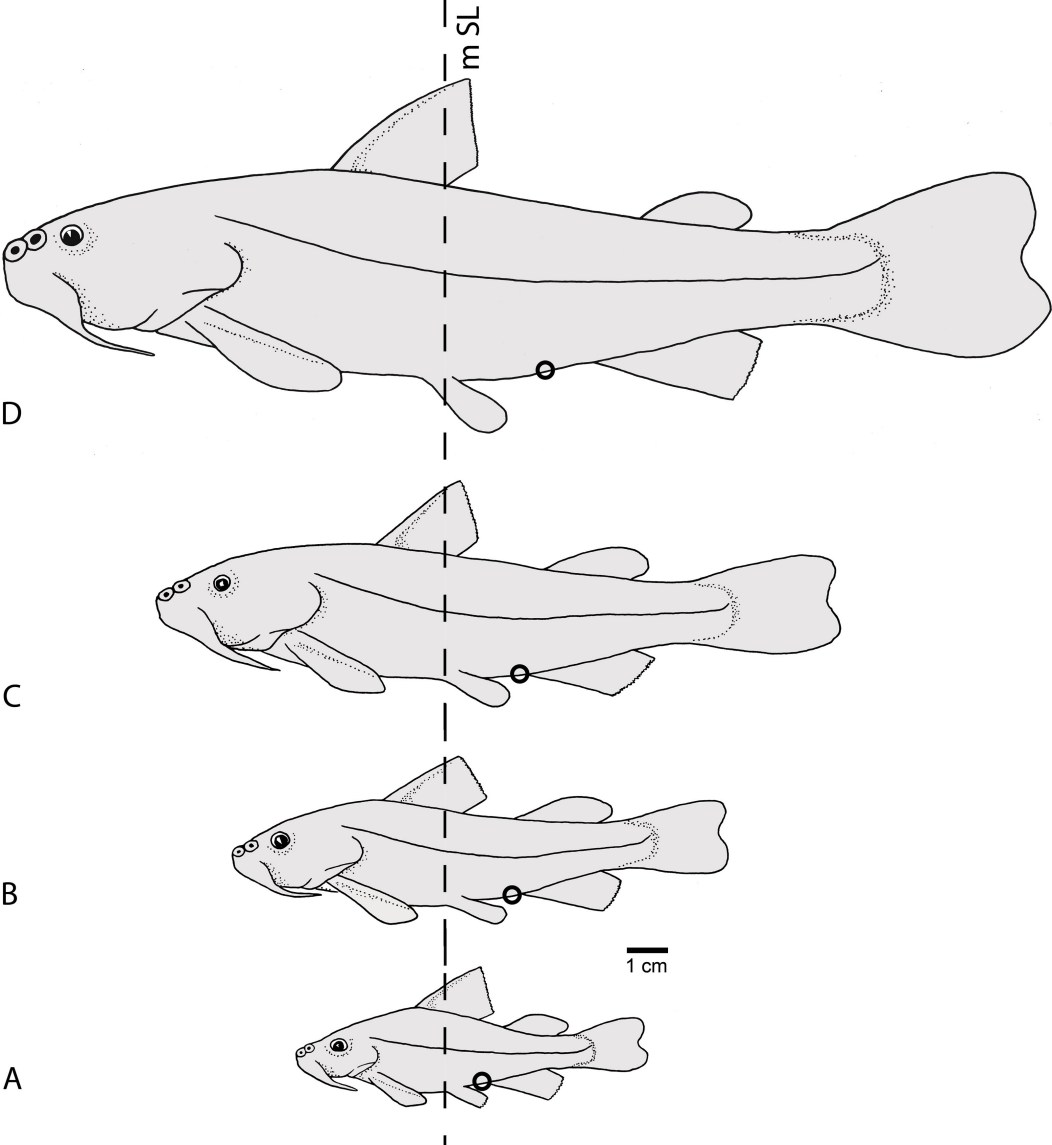
Semidiagrammatic lateral views of *Diplomystes camposensis*. Based on multiple specimens representing the intraspecific ontogenetic variability of body including changes in size, shape, and position of fins and changes in position of anus and urogenital pore; (A) specimens of 50–72 mm SL; (B) specimens of 90–110 mm SL; (C) specimens of 140–150 mm SL; and (D) specimens of 172–220 mm Sl. For comparative purposes, a vertical line is set at mid-length of SL (mSL). A black small circle denotes the position of anus and urogenital pore.

**Figure 10:**
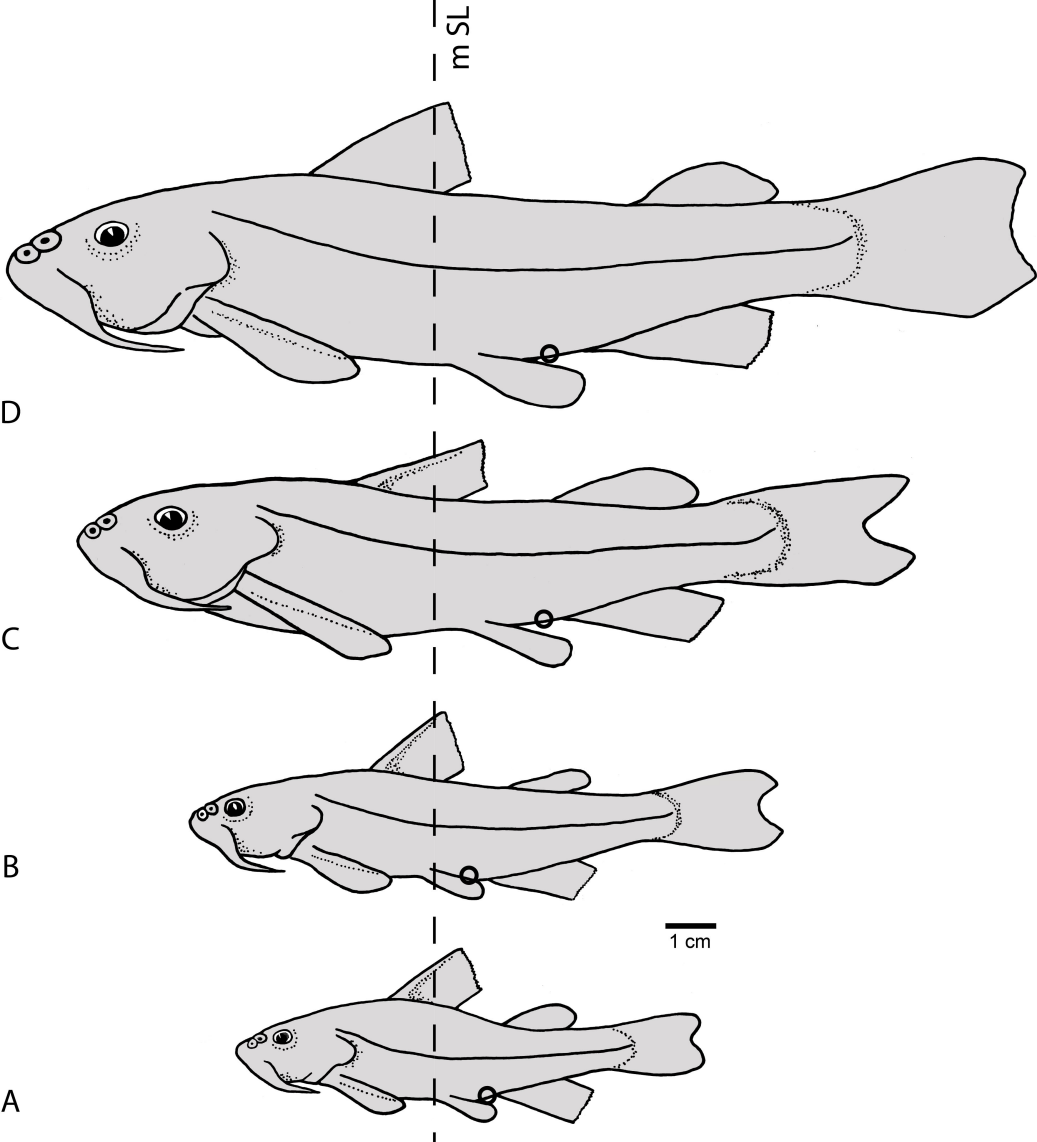
Semidiagrammatic lateral views of *Diplomystes camposensis*. Based on multiple specimens representing the intraspecific ontogenetic variability of body shape including changes in size, shape, and position of fins and changes in position of anus and urogenital pore; (A) specimens of 50–72 mm SL; (B) specimens of 90–110 mm SL; (C) specimens of 140–150 mm SL; and (D) specimens of 172–220 mm SL. For comparative purposes, a vertical line is set at mid-length of SL (mSL). A black small circle denotes the position of anus and urogenital pore.

**Figure 11:**
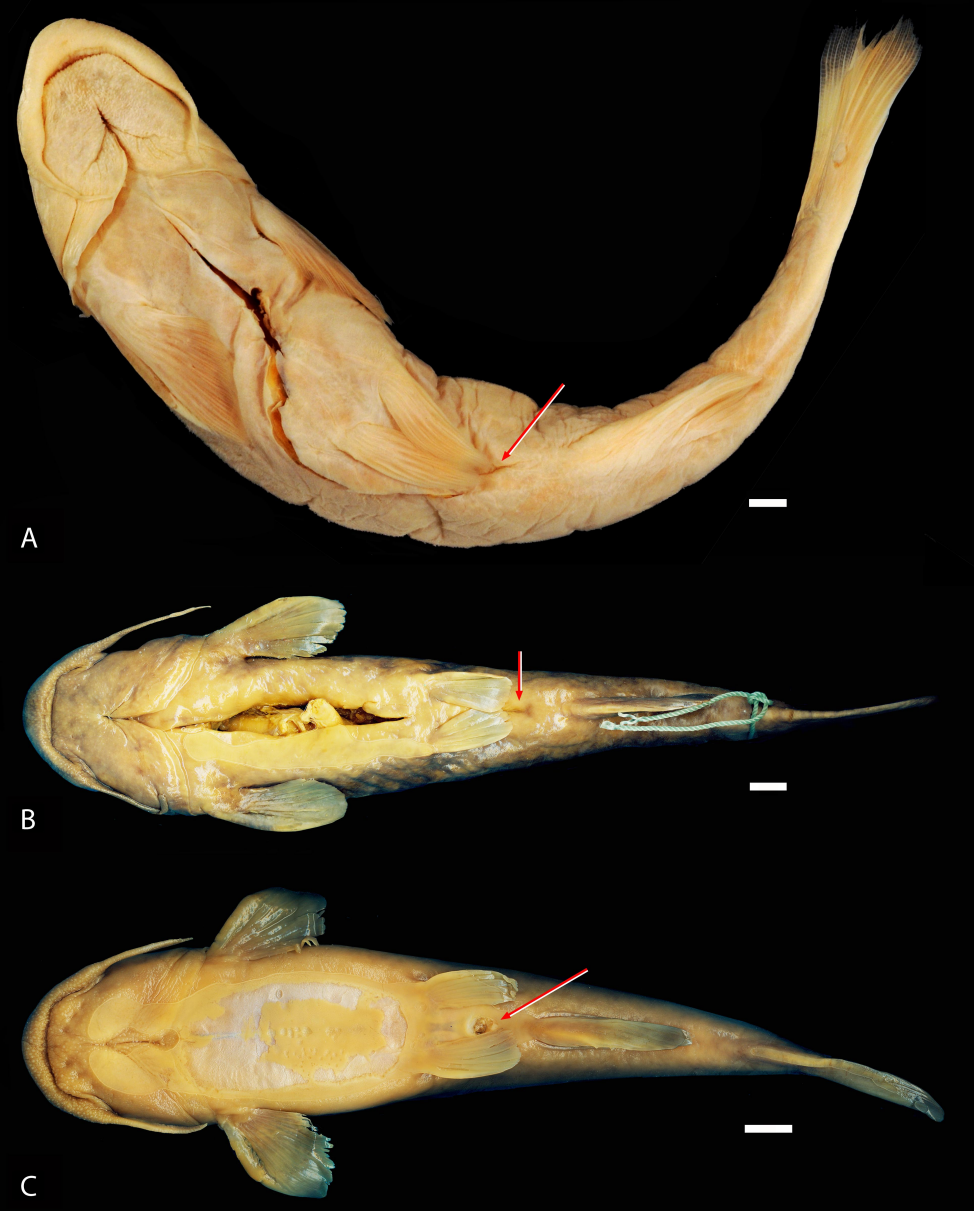
Ventral views of *Diplomystes* species. (A) *Diplomystes chilensis*, MCZ 8290, 153.5 mm SL, Maipo Basin; photograph courtesy of MCZ; all copyrights reserved; (B) *Diplomystes nahuelbutaensis*, LBUCH 010391, 211.1 mm SL, Bío-Bío Basin; photograph courtesy of K Sturm; (C) *Diplomystes camposensis*, IZUA 3303a, 165.9 mm SL, Valdivia Basin; red arrows on the photographs denote the position of anus and urogenital pore; scale bars = 1 cm.

Throughout growth, changes in the distance between fins and the length of the fins in specimens of *Diplomystes nahuelbutaensis* (Figs. 9A–9D) and *D. camposensis* (Figs. 10A–10D) are observed. It is almost impossible to distinguish both species when the fishes are small in size (under 140 mm SL), but after sexual maturity in *D. nahuelbutaensis*, the separation between pelvic and anal fins increases progressively and the anus and urogenital pore are posterior to the distal margins of the pelvic fins (Figs. 9C–9D; 11B). The posterior margins of the pelvic fins in *D. camposensis* stay near the anal fin so that the position of anus and urogenital pore is between both pelvic fins (Figs. 10C–10D, 11C). A similar pattern is observed in *Diplomystes incognitus* sp. nov. The position of the anus and urogenital pore is just posterior to the posterior margins of the pelvic fins in *D. chilensis* (Fig. 11A). The anus and urogenital pore are between the posterior tips of the pelvic fins in *Olivaichthys viedmensis* from Baker River.

The fins differ in shapes. The caudal fin has slightly rounded dorsal and ventral margins and a small notch at the posterior margin in the largest *D. nahuelbutaensis* (Figs. 3B, 4C, 9C–9D). However, in some specimens, the notch is absent in *D. nahuelbutaensis*. The fin is more elongate, with somewhat acuminate dorso- and ventro-posterior margins, and a marked triangular notch in *D. camposensis* (Figs. 3C, 4D, 10C–10D). The shape of the caudal fin of large individuals of *D. chilensis* (Figs. 3A, 4A) is more similar to that of *D. camposensis* than to *D. nahuelbutaensis*. The position of the adipose fin becomes closer to the dorsal margin of the caudal fin in the largest individuals of *D. camposensis*, whereas in *D. nahuelbutaensis* both fins remain distinctively separated. The size of the eyes, which is proportionally large in small specimens of *D. nahuelbutaensis* and *D. camposensis*, becomes larger in *D. camposensis* through growth, and remains small in *D. nahuelbutaensis*. According to our observations, external differences in pelvic fin shape, as well as its position relative to the anus and urogenital pore, are reached in large specimens over 150 mm SL. Sexually mature ovaries and testes have been observed in individuals of 110 mm SL and above in *D.  nahuelbutaensis* (Vila, Contreras & Fuentes, 1996). Such size (110 mm TL) is relatively small in comparison with the largest sizes recorded for *D. nahuelbutaensis* (300 mm TL; Vila, Contreras & Fuentes, 1996) and for *D. camposensis* (242 mm TL; material studied by Curoto (2015) and measured by us).

### Analysis and discussion of some morphological features

#### Skin

The skin is one of the most distinctive characters of Diplomystidae (Arratia, 1987; Arratia & Huaquín, 1995). Histological and SEM studies of the skin of *Diplomystes* and *Olivaichthys* have shown that the skin is characterized by a high number of taste buds all the over the body, including fins and inside of the mouth, and different pit-lines represented by a few, large neuromasts. However, the middle pit-line trunk is represented by a line of small neuromasts along the mid-flank.

**Figure 12:**
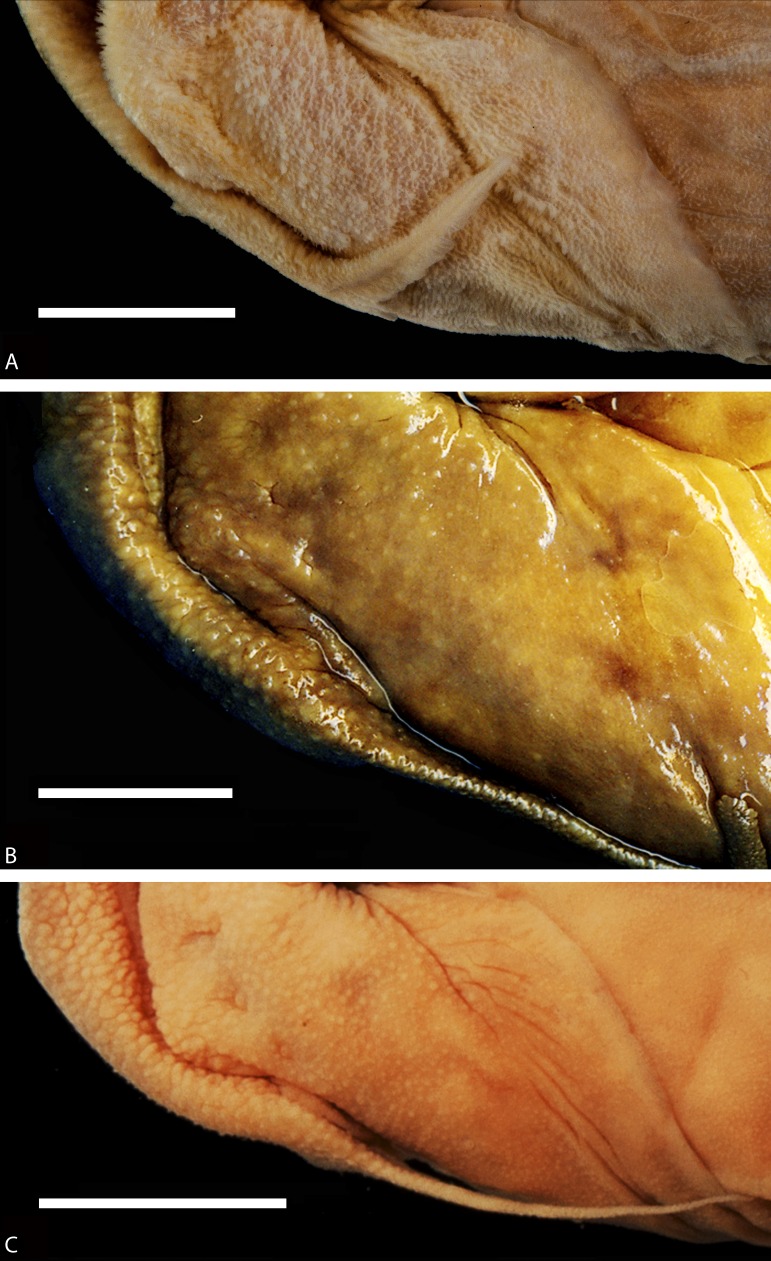
Enlargement of a section of the heads (in ventral views) of diplomystids illustrating the papillae covering the skin of lips, barbels, and gular region. (A) *Diplomystes chilensis*, MCZ 8290; photograph courtesy of MCZ; all copyrights reserved; (B) *Diplomystes nahuelbutaensis*, LBUCH 010391; (C) *Diplomystes camposensis* IZUA 3303a; scale bars = 1 cm.

**Figure 13:**
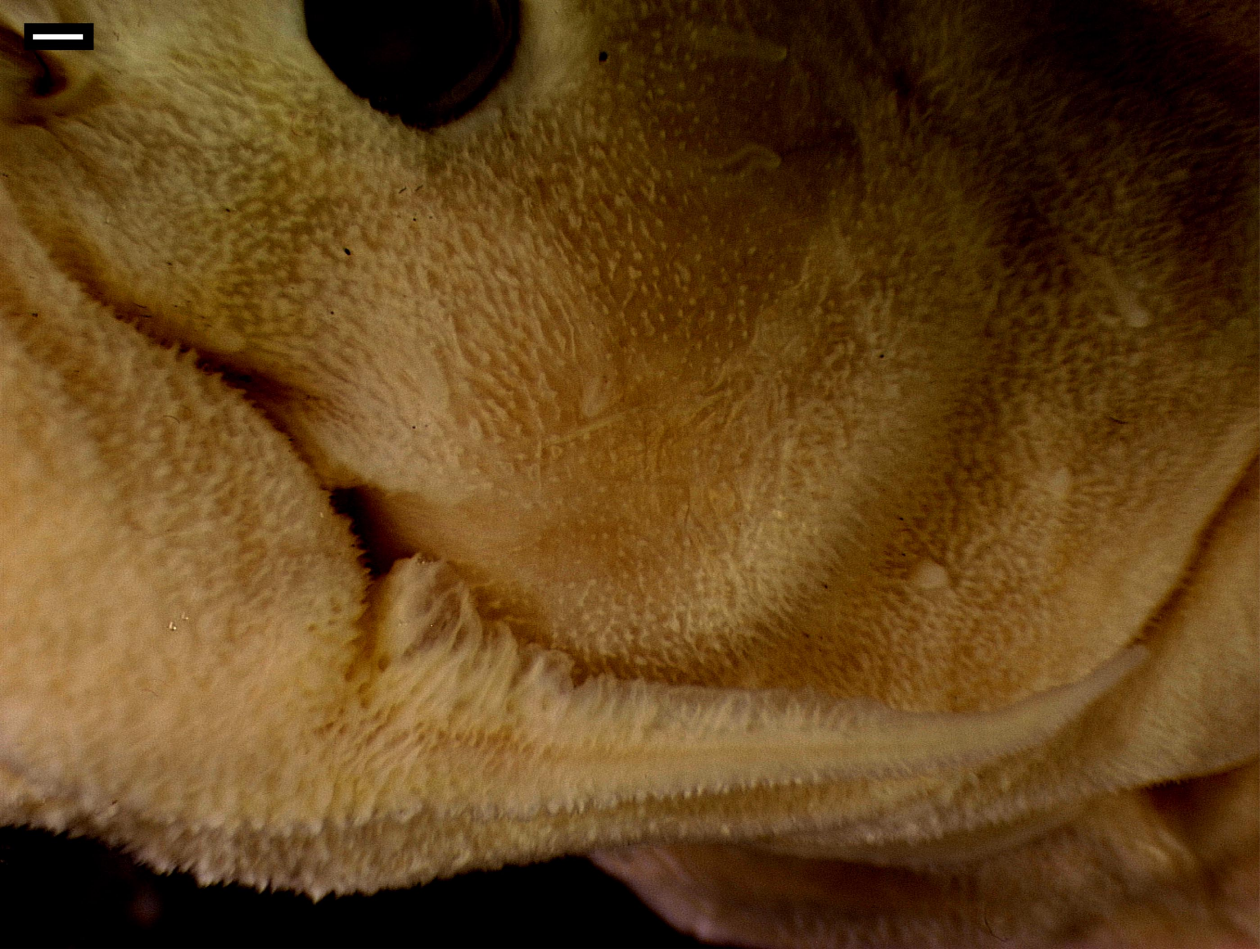
Enlargement of a section of the head (in lateral view) illustrating the papillae of *Diplomystes chilensis*, MCZ 36195. Photograph courtesy of MCZ; all copyrights reserved; scale bar = 1 mm.

**Figure 14:**
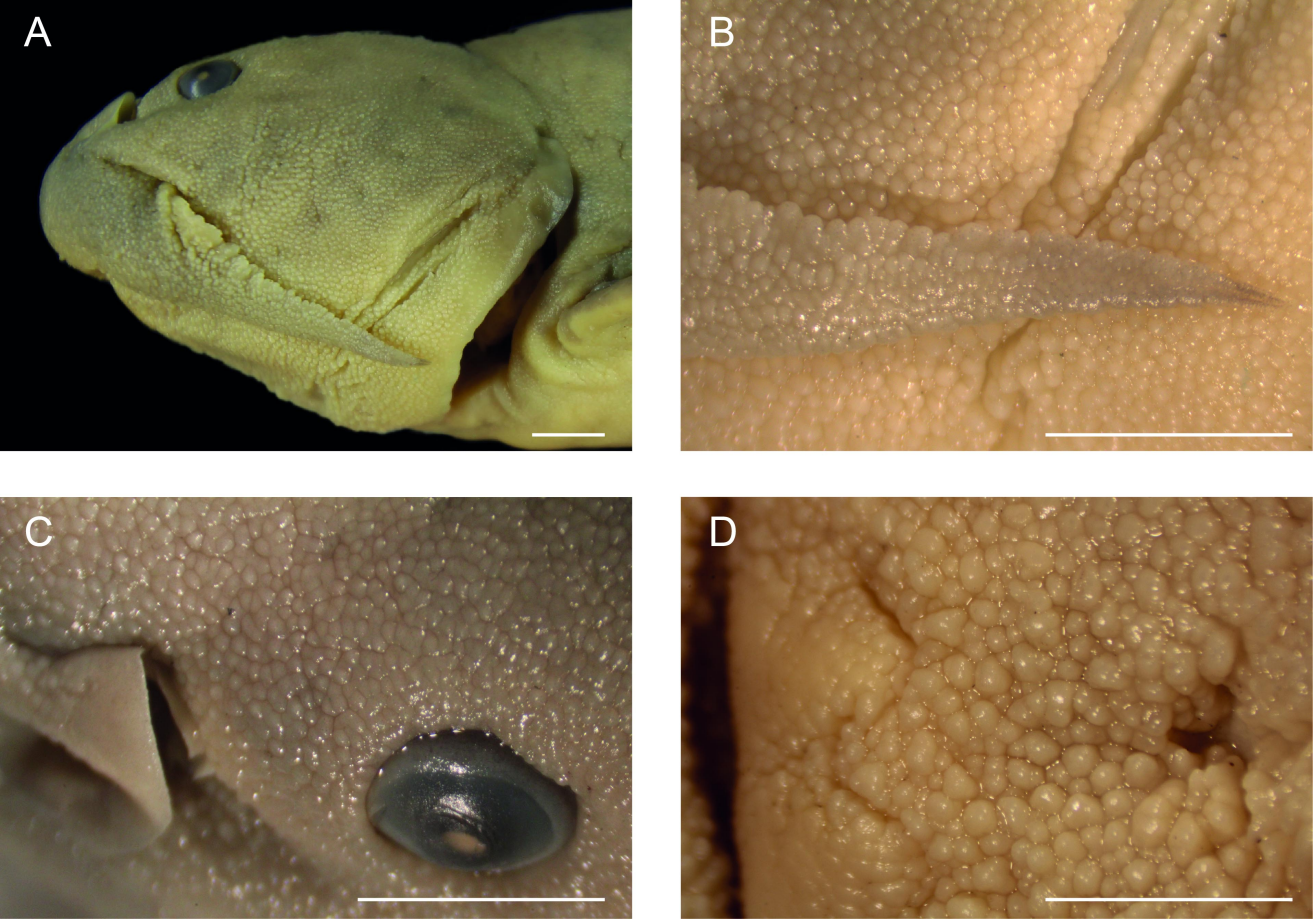
Skin surface of *Diplomystes incognitus* sp. nov., MNHNCL ICT 7538a. (A) head in lateral view; (B) details of the skin surface of distal part of barbel; (C) detail of skin surface around eye and posterior nostril; (D) detail of gular skin surface just posterior to ventral lip; scale bars = 10 mm.

The skin of diplomystids is covered by a variable number of papillae (depending on the age/size) carrying different types of receptors, especially taste buds. Young and juvenile diplomystids have a smooth surface that develops a variable number of papillae during ontogeny. Thus, the descriptions (and illustrations) of the skin presented here correspond to large specimens. *Diplomystes chilensis* is unique among catfishes in the presence of long papillae that give the impression of a “hairy” skin (Figs. 12A, 13). The only known description of a fresh specimen of *D. chilensis* presents it as having its entire body covered by long, gray “hairs” that become slightly brownish toward the caudal region and whitish in the ventral region (Leybold, 1859). We have observed long papillae along the whole body, and they are longer in the barbels (Fig. 13) and flanks, close to the lateral line pathway. Similar long papillae have not been observed in any of the studied diplomystids (compare Figs. 12A, 13 with Figs. 12B–12C and 14) collected south of Maipo Basin (Fig. 2), the type locality of *D. chilensis*.

The papillae of *Diplomystes nahuelbutaensis* (Fig. 12B), *D. camposensis* (Fig. 12C), *Diplomystes incognitus* sp. nov. (Fig. 14), and *Olivaichthys viedmensis* from Baker River are rounded and shorter than in *D. chilensis*, but their distribution differs among the three species.

The skin of the body and mouth of *D. camposensis* is densely covered by papillae that are especially conspicuous on the lips, barbels, and gular region (Fig. 12C). Fewer papillae are present in *D. nahuelbutaensis* (Fig. 12B) and *O. viedmensis*, especially on the dorsal region of the head and flanks. When the skin of these catfishes was observed under SEM (Arratia & Huaquín, 1995), the skin of *D. camposensis* has the unique presence of a reticulated pattern that has not been observed in other species. The skin of *Diplomystes incognitus* sp. nov. (Fig. 14) is characterized by the presence of large and rounded papillae all over the body, that give the skin a “blackberry” or verrucose aspect. The amount of papillae (and size) is so great that the pores of the sensory canals and pit-lines are concealed.

#### Neuromast lines

Although the presence of superficial neuromast lines may be obscured by the development of the papillae, we have gathered new information that may aid in species’ identification. For instance, the rostral line is placed anterior to the anterior nostril, close to the anterior margin of the snout in *Diplomystes chilensis* and *D. camposensis*, whereas in *D. nahuelbutaensis* the anterior nostril is placed almost at the anterior margin of the snout so that the rostral line is placed anteriorly, on the anterodorsal curvature of the snout. The anterior nostril is relatively distant to the anterior margin of the snout in *Diplomystes incognitus* sp. nov., but the rostral line cannot be identified among the rounded papillae covering the region.

**Figure 15:**
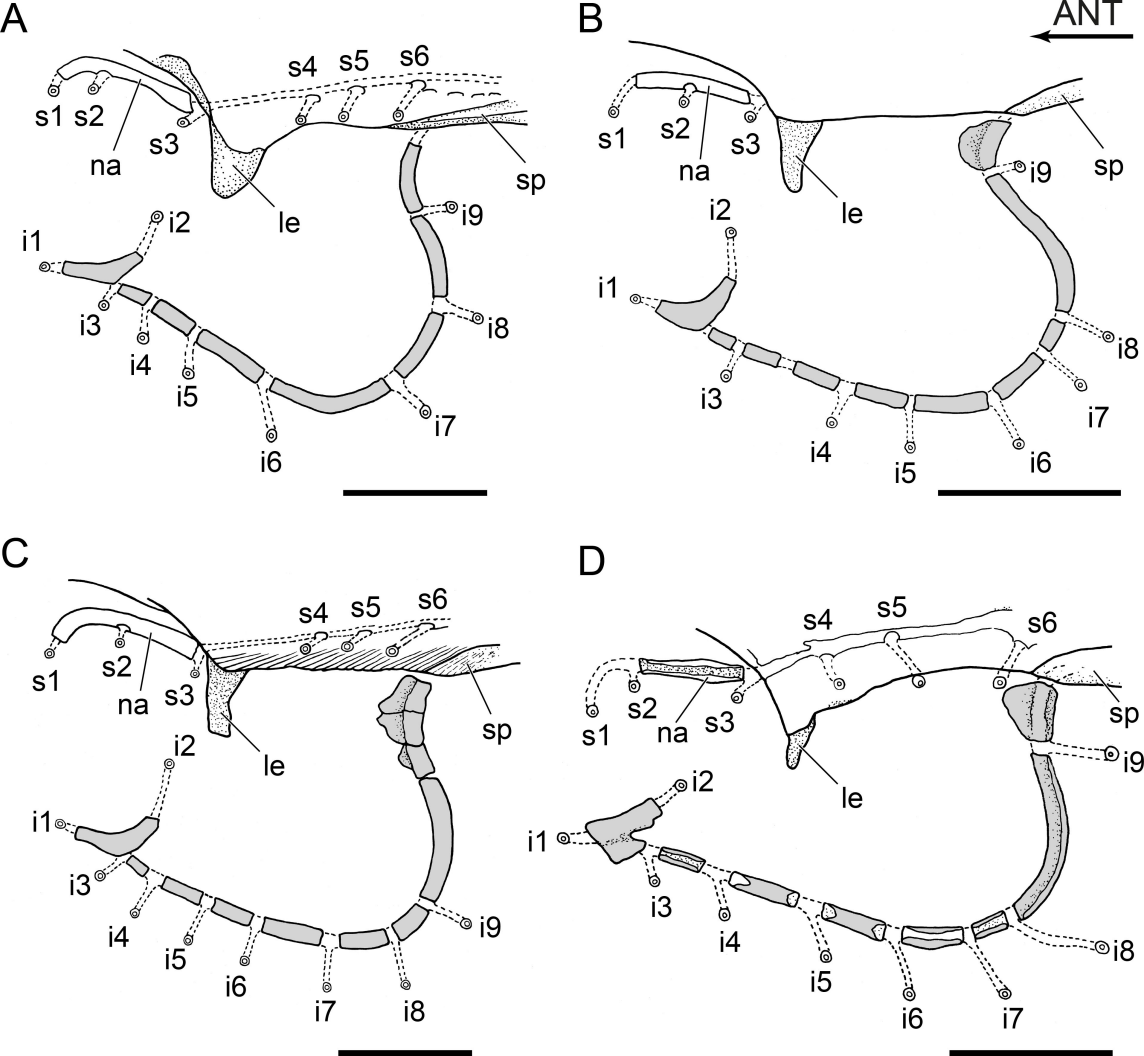
Diagrams of a section of supraorbital sensory canal and its pores and of infraorbital canal and pores and associated bones of *Diplomystes* species. (A) *Diplomystes chilensis*, MCZ 8290, 151 mm SL, Maipo Basin; (B) *Diplomystes incognitus* sp. nov., MNHNCL ICT 7540, 94.7 mm SL, Itata Basin; (C) *D. nahuelbutaensis*, LBUCH 010391, 140 mm SL, Bio-Bio Basin; (D) *D. camposensis*, IZUA 4500, 130 mm SL, Valdivia Basin; scale bars = 5 mm. Abb.: ANT, anterior; i1-9, pores 1–9 of infraorbital canal opening to the skin surface; le, lateral ethmoid; na, nasal bone; sp, sphenotic; s1-6, pores of the supraorbital canal opening to the skin surface.

#### Cephalic sensory canals and main lateral line

The cephalic sensory canal system, including sensory tubules and pores opening to the skin surface, is similar among the species of *Diplomystes* and it has been illustrated for the three species (Arratia, 1987). However, newly observed differences are reported here and we present new information on *Diplomystes incognitus* sp. nov. and *Olivaichthys viedmensis* from the Baker River.

The sensory branches and pores of the supraorbital canal do not differ in position or number among species of *Diplomystes*. The sensory tubules of the infraorbital and preopercular canals are elongate, and their pores open to the skin surface relatively far from the trajectory of the main canal. Although the pores are easily observed in *Diplomystes chilensis* and *D. camposensis*, they are inconspicuous in *D. nahuelbutaensis*, *Diplomystes incognitus* sp. nov., and *Olivaichthys viedmensis* from the Baker River. The number of branches of the infraorbital canal (9) is identical for the species of *Diplomystes* as illustrated in Fig. 15; however, there are differences in the position of the nine branches of the infraorbital canal among the three species. Whereas in *D. chilensis* pores 4–9 are regularly distributed in the ventral and ventroposterior regions of the canal, the distribution of pores 7–9 is different in the three species and in *Diplomystes incognitus* sp. nov. (see Figs. 15A–15D). All pores are concentrated predominantly in the ventral region of the infraorbital canal with pore 9 emerging at the posteroventral corner (and occasionally between the last infraorbital bone and cranial bones) in *D. nahuelbutaensis*, and pore 8 emerging at the posteroventral corner and pore 9 emerging at the posterodorsal region of the canal in *D. camposensis*. Additionally, there are differences in the number and position of the infraorbital bones in the three species, as shown in Figs. 15A–15D (the antorbital (Arratia, 1987; Arratia & Huaquín, 1995) is included in the count of the infraorbital bones). Among these, *D. chilensis* has seven or eight infraorbital bones, whereas *D. nahuelbutaensis* has nine or ten, but the dorsalmost infraorbital bone is formed by fusion or partial fusion of two or three bones (Fig. 15C). *Diplomystes camposensis* has eight or nine infraorbitals, but the most posterodorsal one is not a compound bone. *Diplomystes incognitus* sp. nov. has 10 infraorbitals, as in *D. nahuelbutaensis*, but a dorsalmost compound bone is absent (Fig. 15B). The posterior part of the circumorbital series is formed mainly by a narrow, elongated infraorbital bone in *Diplomystes incognitus* sp. nov., *D. nahuelbutaensis*, and *D. camposensis*, yet it is comparatively shorter in *D. nahuelbutaensis*.

**Figure 16:**
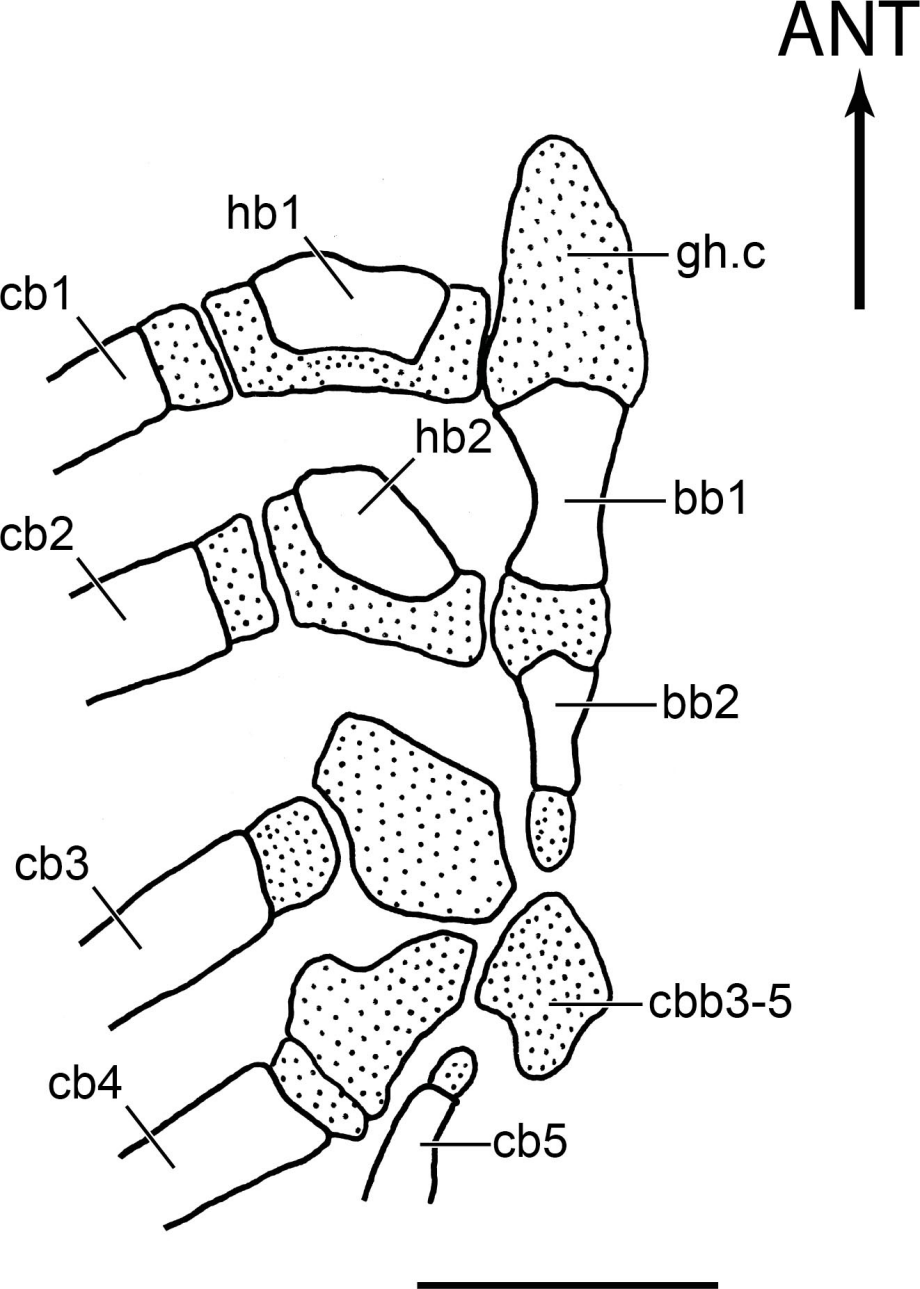
Section of the branchial arches of *Olivaichthys viedmensis*. Note the elongate glossohyal cartilage of a young specimen (modified from Arratia, 1987); scale bar = 1 mm. Abb.: ANT, anterior; bb1–2, basibranchials 1-2; cb1–5, ceratobranchials 1–5; cbb3-5, cartilaginous basibranchial 3-5; hb1–2, hypobranchials 1–2.

The main lateral line and the middle trunk line of neuromasts are continuous along the flank in species of *Diplomystes*. However, *Olivaichthys viedmensis* from Baker River has an interrupted lateral line (see Fig. 4E) between the dorsal and anal fins, and the middle trunk line is complete and composed of small neuromasts.

#### Vomerine tooth plates

The presence of two tooth plates is a condition of young diplomystid specimens. From this early ontogenetic stage, the tooth plates may stay separated during growth (e.g., *D. camposensis* and *D. incognitus* sp. nov.) or become fused (e.g., *D. chilensis* and *D. nahuelbutaensis*). Since this process involves a fusion of elements, some large specimens still show both tooth plates partially fused. In these specimens, this condition has been counted as one element. Comparisons among 19 individuals of over 100 mm SL of *D. chilensis* reveal that one vomerine tooth plate is found in 15 specimens (79%) and two tooth plates are present in four specimens (21%). In contrast, two vomerine tooth plates are found in 19 specimens (83%) of a total of 23 large individuals of *D. camposensis* and only one tooth plate is present in four specimens (17%). Only one tooth plate is present in 23 individuals (90%) among 25 large specimens of *D. nahuelbutaensis* and two tooth plates are present in two specimens (10%). Among the largest available specimens (four) of *Diplomystes incognitus* sp. nov., three are present with two tooth plates. Two tooth plates are also present in the two larger specimens of *Olivaichthys viedmensis* from the Baker River.

#### Glossohyal

The branchial arches in Diplomystidae have been described and/or illustrated by Arratia (1987), Arratia (2003), Azpelicueta (1994), and Mabee et al. (2011). It is not our intention to discuss the branchial arches of diplomystids but to report an interesting feature that we have observed during this study.

**Figure 17:**
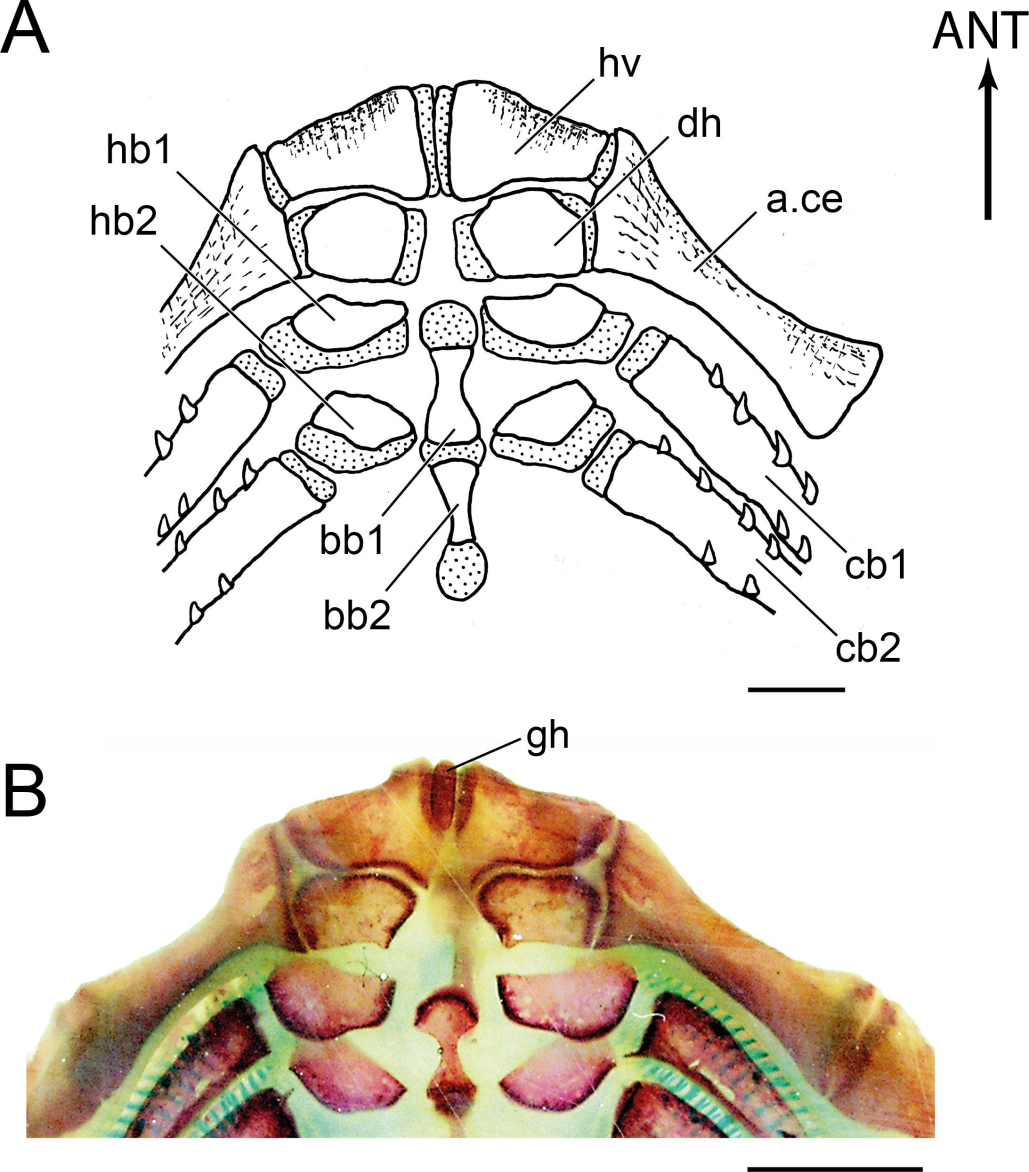
Section of the hyoid and branchial arches in *Diplomystes nahuelbutaensis*. (A) Anterior region of the branchial apparatus (LBUCH 010391), illustrating the common condition of diplomystids; scale bar = 5 mm; (B) anterior region of the branchial apparatus and glossohyal ossification (LBUCH 010391); scale bar = 5 mm. Abb.: a.ce, anterior ceratohyal; ANT, anterior; bb1–2, basibranchials 1–2; cb1–2, ceratobranchials 1–2; gh, glossohyal; hb1–2, hypobranchials 1–2; dh, dorsal hypohyal; vh, ventral hypohyal.

An elongated median cartilage extending anteriorly to the hypohyal region was illustrated in young specimens of *Olivaichthys viedmensis* (Arratia, 1987; Fig. 16 herein). The position of this cartilage in front of the first ossified basibranchial and its dorsal extension to the hypohyal region could be interpreted as a glossohyal cartilage. Because the absence of a bony glossohyal is considered a synapomorphy of Siluriformes, this cartilage was not labeled by Arratia (1987). We agree with the common interpretation that the absence of the glossohyal (Fig. 17A) is a feature characterizing the order, thus, we were surprised to find a small median bone in front of the basibranchials and above the dorsal hypohyals in *D. nahuelbutaensis* (Fig. 17B). We interpret this bone as a glossohyal that may be an atavism that is occasionally present in diplomystids.

More primitive ostariophysans, such as gonorynchiforms and cypriniforms, have a well-developed glossohyal.

#### Pelvic radial

A free cartilaginous pelvic radial, independent of the posterior articular surface of the basipterygium and of its medial posterior cartilaginous process, was described and illustrated for *Olivaichthys viedmensis* by Arratia (1987: Fig. 37B). It was interpreted as a synapomorphy of the genus *Olivaichthys*. However, a cartilage that is part of the articular posterior surface of the basipterygium was interpreted as a pelvic radial in some specimens of *Diplomystes nahuelbutaensis* and *D. camposensis* by Azpelicueta (1994). According to our observations, pelvic radials are independent cartilaginous elements in a few teleosts and extant neopterygians where these elements occur. No independent pelvic radials were observed in any specimen of *Diplomystes* studied herein. We suggest further research on the variability reported for the Argentinean diplomystids (Azpelicueta, 1994).

#### Pores of axillary gland

Members of the family Diplomystidae are characterized by the presence of an axillary gland that is placed just dorsomedial to the insertion of the pectoral spine. It may open to the skin surface by a variable number of pores. *Diplomystes chilensis* presents one to three pores, commonly two in both sides of the body (Arratia, 1987), a count that is distributed as follows among 12 specimens: Two pores in eight specimens (67%), one pore in three specimens (25%), and three pores in one specimen (8%). In contrast, *D. nahuelbutaensis* was reported to have one to three pores, commonly three on each side of the body (Arratia, 1987). The larger sample of specimens studied has changed such information and has provided new findings: two pores in both sides of the body are present in 22 individuals (44%) among a total of 50 examined specimens, the absence of pores is found in 19 specimens (38%), one pore is found in four specimens (8%), and three pores in one or both sides of the body are found in five specimens (10%).

*Diplomystes camposensis* was previously reported as having no pores and/or a single on one side of the body (Arratia, 1987). The new results, based on 57 examined specimens, show slightly more variability with 36 individuals (63%) demonstrating no axillary pores opening to the skin surface, nine specimens (16%) possessing either one pore on one or both sides of the body, 11 specimens (19%) with two pores in either one or both sides of the body, and one specimen (2%) with three pores. *Diplomystes incognitus* sp. nov. shows no axillary pores opening to the surface in the seven studied specimens, but four pores are found on one side of the body in one individual studied. The two large specimens of *Olivaichthys viedmensis* from the Baker River show no axillary pores, similarly to the condition observed in five larvae. Pores of the axillary gland are present at all growth stages. Therefore, ontogenetic variability is refuted for all species.

### Revised diagnoses of species of *Diplomystes*

**Table:** 

***Diplomystes chilensis* (Molina, 1782)**
(Figs. 3A, 4A, 11A, 12A, 13, 15A)

**Diagnosis** (emended from Arratia, 1987). Diplomystid that is distinguished from all congeners by the possession of “hairy-like” skin, covered with long, simple or lobulated papillae (vs. granulose skin with short papillae); acuminated pectoral fins (vs. distally rounded fins); fused anterior processes of autopalatine (vs. separated processes); lateral line enclosed by ossified, tube-like ossicles along the whole flank (vs. few ossicles at the anterior third of flank in other *Diplomystes*). With few maxillary teeth (8–13, commonly 9) vs. 11–13 (commonly 11) in *D. nahuelbutaensis*, 12–19 in *D. camposensis* (commonly 15), and 7–9 in *Diplomystes incognitus* sp. nov. *Diplomystes chilensis* can further be differentiated from *Diplomystes incognitus* sp. nov. as having a less deep dorsal fin (15.5–18.7% of SL vs. more than 18.7%).

**Table:** 

***Diplomystes nahuelbutaensis* Arratia, 1987**
(Figs. 3B, 4C, 8A–8C, 9A–9D, 11B, 12B, 15C, 16B–16C)

**Diagnosis** (emended from Arratia, 1987). Diplomystid that is distinguished from all congeners by the possession of a skin covered with short, round papillae, sparsely distributed on the dorsal region of the head and flanks; anterior nostril placed anterodorsally (vs. dorsally placed); rostral pit-line placed on anterodorsal margin of snout (vs. dorsally placed); anterior and posterior nostrils surrounded by narrow fold of skin; thus, posterior nasal opening may be partially or totally exposed (vs. nostrils surrounded by large skin fold); one (fused) vomerine tooth plate in most adults (vs. two separate tooth plates); 11–13 maxillary teeth, commonly 11 (vs. 8–13 in *D. chilensis*, 12–19 in *D. camposensis*, and 7–9 in *Diplomystes incognitus* sp. nov.); dentary with a ventral bony flange close to the symphysis (vs. absence of flange); lateral line surrounded by few (less than five) ossified tube-like ossicles in adults; urogenital pore and anus placed in between the posterior tips of pelvic fins and anal fin (vs. urogenital pore and anus placed in between pelvic fins). It can be further differentiated from *D. chilensis* and *D. camposensis* by the inconspicuous cephalic sensory pores especially in adults (vs. clearly visible cephalic sensory pores); and from *Diplomystes incognitus* sp. nov. and *D. camposensis* by having two or occasionally three pores of the axillary gland (vs. no pores or occasionally one).

**Table:** 

***Diplomystes camposensis*** **Arratia, 1987**
(Figs. 3C, 4D10A–10D, 11C, 12C, 15D)

**Diagnosis** (emended from Arratia, 1987). Diplomystid that is distinguished from all congeners by the possession of the skin of head, body, and fins densely covered by round, short papillae; maxilla with 12–19 teeth, commonly 15 (vs. 8–13 in *D. chilensis*, 7–9 in *Diplomystes incognitus* sp. nov. and 11–13 in *D. nahuelbutaensis*); short nasal bone situated between supraorbital sensory canal pores 2 and 3 (vs. nasal extending beyond supraorbital pore 2); elongate autopalatine, its facets for lateral ethmoid and cartilage joining with mesethmoid, vomer, lateral ethmoid, and orbitosphenoid along first third of bone (vs. half its length in other *Diplomystes*); and, absent pores of axillary gland with occasionally one or two on one or both sides of body (vs. two or three pores).

**Table:** 

**Figure 18:**
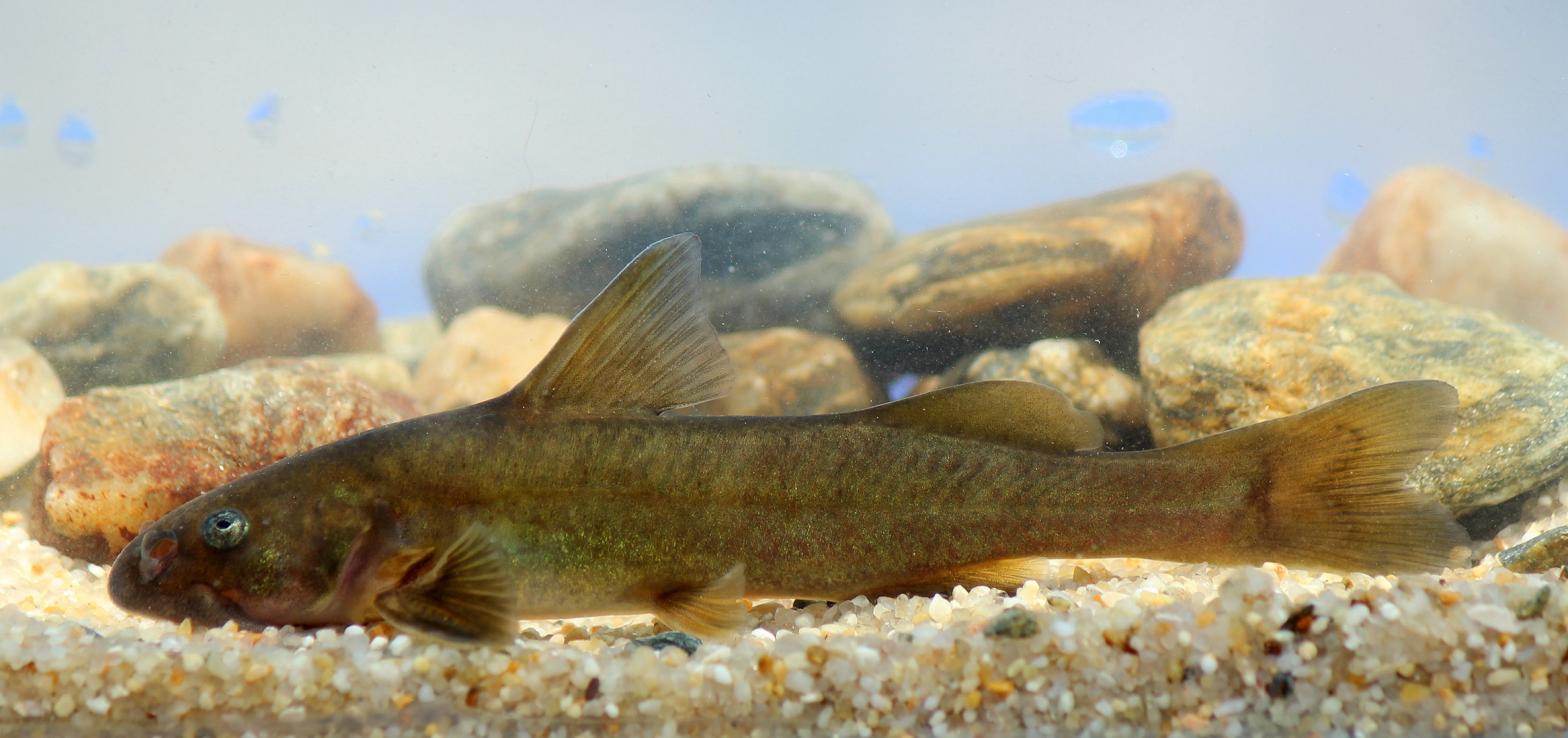
*Diplomystes incognitus* sp. nov. in a recreation of its natural environment. Young individual, ca. 93 mm SL, from Ñuble River at Nahueltoro Bridge, Itata Basin.

***Diplomystes incognitus* sp. nov.**
(Figs. 4B, 8C, 14A–14D, 15B, 18)
*Diplomystes chilensis*: Arratia, Chang & Rojas, 1981: 34 (in part)
*Diplomystes chilensis*: Arratia, 1982: Figs. 7A–7B
*Diplomystes chilensis*: Arratia, 1983: 222, tbs. 5–6 (in part)
*Diplomystes* sp.: Arratia, 1987: 34
*Diplomystes* spec.: Arratia & Huaquín, 1995: 44–46
*Diplomystes* aff. *chilensis*Arratia & Huaquín, 1995: 46–50
*Diplomystes* cf. *chilensis*: Muñoz-Ramírez et al., 2014: tb. 4.
*Diplomystes* cf. *chilensis*: Muñoz-Ramírez, Victoriano & Habit, 2015: genetic structure

**Diagnosis.** Diplomystid that is distinguished from all congeners by the possession of the skin of head, body, and fins densely covered by round, short papillae giving the skin a blackberry-like or verrucose aspect in large individuals; with a short head, slightly squarish and as long as broad (versus slightly longer more triangular-shaped head); high dorsal fin, ca. 20% of SL (range 17–25%) and triangularly-shaped (versus slightly rhomboidal); maxilla with 7–9 teeth (vs. 8–13 in *D. chilensis*, 11–13 in *D. nahuelbutaensis,* and 12–19 in *D. camposensis*); with 10 infraorbital bones, as in *D. nahuelbutaensis*, but the dorsalmost compound bone is absent; urogenital pore and anus placed between posterior tips of pelvic fins as in *D. chilensis* (vs. urogenital pore and anus placed between pelvic fins or in between the distal tips of pelvics and anal fin); and absence of pores of axillary gland with occasionally four on one side of body (vs. two or three pores).

**Type material**. All from Chile: MNHNCL ICT 7538a, holotype, 153.0 mm SL; Melado River, Andean region of Linares, Maule Basin, Región del Maule, 35°43′10″S 71°04′09″W; no other data; [field nr. = PC 010114].—MNHNCL ICT 7538b, paratype, 132.2 mm SL; Melado River, Andean region of Linares, Maule Basin, Región del Maule, 35°43′10″S 71°04′09″W; no other data; [field nr. = PC 010114].—MNHNCL ICT 7539, paratype, 108.2 mm SL; Ancoa River at Chupallar Bridge, Maule Basin, Región del Maule, 35°54′1″S 71°17′03″W; C Andrade; no other data; [field nr. = PC 010408]. —MNHNCL ICT 7540, paratype, 94.7 mm SL; Diguillín River, Itata Basin, Región del Bío-Bío; C  Andrade; no other data; [field nr. = PC 020308].—MNHNCL ICT 7541, 3 paratypes, 35.05–39.50 mm SL; Ñuble River at Nahueltoro Bridge, Itata Basin, Región del Bío-Bío, 36°29′0.6″S 71°45′20.5″W; C Quezada-Romegialli and J  Benavente, February 10, 2016; [field nr. = PC 100216]. —MNHNCL ICT 7542, 1 paratype, 134 mm SL; 1, 134.0 mm SL; Copequén River, Rapel Basin, Región del Libertador Bernardo O’Higgins, 34°14″S 70°55″W; M Arellano & F Camilo; no other data; (=LBUCH 310883 in Arratia & Huaquín, 1995).

**Additional specimens**. CAS 55426, 98.3 mm SL; Loncomilla River, San Javier, Maule Basin, Región del Maule, 35°35′41″S 71°44′58″W; C Eigenmann, March 23, 1919 (previously described as a paratype of *D. nahuelbutaensis* by Arratia, 1987). —KUNHM 19255, 1, 69.4 mm SL; Maule River, Maule Basin, Región del Maule; H Díaz & A Chang, December 2, 1974.—KUNHM 19256, 1, 103.7 mm SL; Tinguiririca River at Los Maques, Rapel Basin, Región del Libertador Bernardo O’Higgins; I. Cid, February, 1976.—LBUCH 011286, 1, 134.0 mm SL; Rapel Reservoir, at Las Balsas, Rapel Basin, Región del Libertador Bernardo O’Higgins, 34°11′29″S 71°27′51″W; L Huaquín & F Camilo, 1986.—LBUCH 031008; 1, 80.8 mm SL; Ancoa River, Maule Basin, Región del Maule, 35°55′28.6″S 71°26′21.9″W; C  Andrade; no other data.

**Etymology**. The specific name *incognitus* is in reference that recognition of the species was obscured by the assumption that *Diplomystes chilensis* also extended south of Maipo Basin.

**Geographical distribution.** In Rapel, Mataquito, Maule, and Itata Basins (Fig. 2).

**Coloration.** The skin of live, young and juvenile individuals (Fig. 18) is greenish in the dorsal part of the head and body and dorsal and adipose fins, with minuscule black and golden spots. The greenish color is also observed irregularly in the tail. The ventral body is of brown-reddish color, with a yellow or creamy colored belly. The lateral aspect of the head is of a gray and reddish color mixture. Large individuals have similar coloration when alive, which turns almost dark red-brown or dark gray-brown at death. Fishes fixed in ethanol become a uniformly gray or brown color.

**Comments**. For morphometric data see Table 1 and for number of branchiostegals, vertebral elements, and fins rays see Table 4.

**Table 4:** Number of branchiostegals, vertebral elements, and fin rays in species of *Diplomystes*. Roman numbers: spines; sp, splint.

	*D. chilensis*	*D. incognitus* sp. nov.	*D. nahuelbutaensis*	*D. camposensis*
Branchiostegals	8–9	8	9–10	9–10
Vertebrae	41–43	40	30–41	40–44
Abdominal vertebrae	14–17	16	15–17	15–17
Caudal vertebrae	23–27	24	23–26	26–27
Ribs	10–12	10	11–13	11–13
Pectoral rays	I+9–10	1+9	I+9	I+9
Pelvic rays	sp+6	sp+6	sp+6	sp+6
Dorsal rays	II+7	II+6–7	II+7	II+7
Dorsal pterygiophores	8	7–8	8	8
Anal rays	12–15	13	14–15	11–15
Anal pterygiophores	10–12	10–11	11–13	12
Caudal rays	47–53	46–50	47–52	52–56
Dorsal procurrent rays	14–18	15	15–18	17–19
Ventral procurrent rays	14–17	12–16	14–18	17–19
Rays of dorsal caudal lobe	23–17	25	24–27	26–28
Rays of ventral caudal lobe	23–26	21–25	23–27	25–28

### Key to diplomystids

**Table:** 

1a - Maxillae without dentition;	Other extant siluriforms	
1b –Maxillae toothed;	Family Diplomystidae	(2)
2 –Long and continuous lateral line; maxilla with two rows of teeth anteromedially, one row posterolaterally; with 8 to less than 20 maxillary teeth (8–19); loss of a separate cartilaginous pelvic radial;	Genus *Diplomystes*	(3)
2b –Lateral line interrupted, divided in two sections; maxilla with more than two rows of teeth anteromedial, two or more rows posterolaterally; with more than 20 maxillary teeth;	*Olivaichthys viedmensis*	
3a –Skin of head, body, and fins densely covered with long, fine, “hairy” papillae; pectoral fin with posterior margin acuminate;	*Diplomystes chilensis*	
3b –Skin with granulose aspect, with rounded papillae; pectoral fin with posterior margin rounded;		(4)
4a –Short and round papillae densely distributed all over the body; straight posterior margin of pelvic fins; posterior margin of pelvic fins near anal fin origin; urogenital pore and anus placed between pelvic fins in large individuals (ca. 150 mm SL and above);	*Diplomystes camposensis*	
4b –Round and short papillae sparsely distributed on head, body and fins; posterior margins of pelvic fins rounded; posterior margin of pelvic fins broadly separated from anal fin origin; urogenital pore and anus in between posterior tips of pelvic fins and anal fin (ca. 150 mm SL and above);	*Diplomystes nahuelbutaensis*	
4c –Round, long papillae densely covering head, body, and fins (verrucose or blackberry-like skin dimpled surface); high dorsal fin (20% of SL; range 17–25%), triangularly-shaped; urogenital pore and anus placed between posterior tips of pelvic fins;	*Diplomystes incognitus* sp. nov.	

## Final Comments

This study illustrates the difficulties in the identification of species of *Diplomystes*, which possess similar external aspects, especially in young and juvenile specimens. Because of this fact, we concentrated our efforts on characters that could identify and separate these species. During this research, it became obvious that sexually immature specimens and specimens below 150 mm SL belonging to different species are very similar in their external morphology. This includes similarities in body proportions, fin shape and size, and a smooth skin with few or no papillae. These external morphological characteristics become significantly different in large *Diplomystes chilensis*, *D. nahuelbutaensis*, *D. camposensis*, and *Diplomystes incognitus* sp. nov., however, not in the morphometric information including body proportions, the ranges of which overlap despite differences in means (Table 1). These findings provide evidence that members of *D. nahuelbutaensis* and *D. camposensis* (Figs. 9 and 10) undergo some significant changes in certain body features throughout growth, especially in the relationships between pelvic and anal fins and the position of the anus and urogenital pore. These changes are unknown in other siluriforms at this present time.

All known specimens of *D. chilensis* are large. No young specimens are available for study. We predict that they are also indistinguishable from the other two species when young. Independent of these assumptions, several morphologies separate the four Chilean species of *Diplomystes* as illustrated in the amended diagnoses and the identification key. Based on our findings, the diplomystids inhabiting rivers from Rapel to Itata Basins (Fig. 2) now are classified in a new species identified here as *Diplomystes incognitus* sp. nov. This group is distinguished by its measurements (Fig. 7), external body shape (Fig. 4B), and morphological characters (e.g., number of maxillary teeth, skin surface) from *D. chilensis* and other species of *Diplomystes* (compare Figs. 12A, 13 and 14A–14D).

A morphological feature of special interest is the skin. The histological structure of the skin of the primitive catfishes, *Diplomystes* and *Nematogenys*, is similar, including the presence of superficial papillae, numerous taste buds, and specific pit-lines formed by a few neuromasts (Arratia & Huaquín, 1995). Though the presence of papillae may be shared by different catfishes, its morphology and distribution is distinct and separate species of Diplomystidae. *Diplomystes chilensis* has especially long papillae. In contrast, *Diplomystes incognitus* sp. nov. has a verrucose or blackberry-like, dimpled skin surface with long and rounded papillae all over its body. *Diplomystes camposensis* has its skin densely covered by short, rounded papillae all over its body, including the fins. Large *D. nahuelbutaensis* and *Olivaichthys viedmensis* have sparsely distributed short, rounded papillae on the body and fins.

The four species of *Diplomystes* (including *Diplomystes incognitus* sp. nov.) have a long, continuous main lateral line that reaches the beginning of the caudal fin (Figs. 4A–4D) and a middle pit-line trunk that follows the main lateral line in its trajectory. In *Olivaichthys viedmensis* from the Baker River, the lateral line canal (but not the middle pit-line trunk) is discontinuous (Fig. 4E). This feature is being reported for the first time and should be further investigated in Argentinean specimens.

After revising the osteological morphology of *Diplomystes*, we did not find significant differences with the previous descriptions (e.g., Arratia, 1987; Arratia, 1992). We did discover in a few specimens an unexpected feature: the presence of an enlarged glossohyal cartilage and its ossification in some specimens. This feature is hypothesized here as a possible atavism after comparison with the generalized condition present in most teleosts including other ostariophysans. Another hypothesis is that it could represent an anomaly. This hypothesis, however, seems unsupported by the otherwise “normal” general aspect of the specimens.

The morphological revision of characters of the species of *Diplomystes*, with the new evidence offered here, strongly support their recognition as valid species. The new species described here is supported by morphological characters (see diagnosis) and by molecular studies (Muñoz-Ramírez et al., 2014). Our results disagree with the proposal that *Diplomystes camposensis* may be a synonym of *D. nahuelbutaensis* according to the molecular results of Muñoz-Ramírez et al. (2014). The distinctness of *D. nahuelbutaensis* and *D. camposensis* is supported by numerous morphological characters (see diagnoses above) and information provided by their karyotypes (including specimens of *D. camposensis* from Toltén and Valdivia Basins). Although the diploid number of chromosomes and chromosomes formulae are comparable, differences in C-banding are present (Campos, Arratia & Cuevas, 1997). According to Muñoz-Ramírez et al. (2014) the populations found in the Bío-Bío Basin would represent a new species, but not *D*. *nahuelbutaensis*. Our findings do not support such a claim. We have expanded our analysis to include large specimens collected from the upper part of the Bío-Bío Basin, a region not sampled by those authors. Muñoz-Ramírez et al. (2014) proposed a multi-species hypothesis concerning diplomystids (e.g., individuals from Toltén Basin would not correspond to *D. camposensis* but to a new sister species of *D. nahuelbutaensis*). We consider this hypothesis premature; it should be investigated further, incorporating diplomystid populations living in between Bío-Bío and Valdivia Basins, a region including the Toltén Basin, which is incompletely sampled.

## Additional Information and Declarations

### Data Availability

The following information was supplied regarding data availability: Raw data used in statistical analyses can be found at GitHub: https://github.com/clquezada/Diplomystes/tree/master/Arratia%20%26%20Quezada-Romegialli%20PeerJ%202016.

### New Species Registration

The following information was supplied regarding the registration of a newly described species: *Diplomystes incognitus* sp. nov. : urn:lsid:zoobank.org:act:98380433-109C-49AD-8459-BA2E50F13817, Publication LSID: urn:lsid:zoobank.org:pub:D22D3881-ED19-470C-8BAF-0CA306EA073B.

## References

[ref-1] Arratia G (1982). El esqueleto caudal de los peces Siluriformes y sus tendencias evolutivas (Fam. Diplomystidae and Trichomycteridae). Boletín del Museo Nacional de Historia Natura.

[ref-2] Arratia G (1983). Preferencias de habitat de peces siluriformes de aguas continentales de Chile (Fam. Diplomystidae y Trichomycteridae). Studies on Neotropical Fauna and Environment.

[ref-3] Arratia G (1987). Description of the primitive family Diplomystidae (Siluriformes, Teleostei, Pisces): morphology, taxonomy, and phylogenetic implications. Bonner Zoologische Monographien 24.

[ref-4] Arratia G (1992). Development and variation of the suspensorium of primitive catfishes (Teleostei: Ostariophysi) and their phylogenetic relationships. Bonner zoologische monographien, 32.

[ref-5] Arratia G, Arratia G, Kapoor BG, Chardon M, Diogo R (2003). Catfish head skeleton. An overview. Catfishes.

[ref-6] Arratia G, Chang A, Rojas G (1981). Géneros de peces de aguas continentales de Chile. Museo Nacional de Historia Natural, Chile Publicación Ocasional.

[ref-7] Arratia G, Huaquín L (1995). Morphology of the lateral line system and of the skin of diplomystid and certain primitive loricarioid catfishes and systematics and ecological considerations. Bonner Zoologische Monographien, 36.

[ref-8] Arratia G, Menu-Marque S (1981). Revision of the freshwater catfishes of the genus *Hatcheria* (Siluriformes, Trichomycteridae) with commentaries on ecology and biogeography. Zoologischer Anzeiger.

[ref-9] Arratia G, Schultze H-P (1992). Reevaluation of the caudal skeleton of certain actinopterygian fishes: III. Salmonidae. Homologization of caudal skeletal structures. Journal of Morphology.

[ref-10] Azpelicueta MM (1994). Three East-Andean species of *Diplomystes* (Siluriformes: Diplomystidae). Ichthyological Exploration of Freshwaters.

[ref-11] Beltrán-Concha M, Muñoz-Ramírez C, Ibarra J, Habit E (2012). Análisis de la dieta de *Diplomystes* (Siluriformes: Diplomystidae) de Chile. Gayana (Concepción).

[ref-12] Borcard D, Gillet F, Legendre P (2011). Numerical ecology with R.

[ref-13] Britz R, Kakkassery F, Raghaven RF (2014). Osteology of *Kryptoglanis shajii*, a stygobitic catfish (Teleostei: Siluriformes) from Peninsular India with a diagnosis of the new family Kryptoglanidae. Ichthyological Exploration of Freshwaters.

[ref-14] Campos H, Arratia G, Cuevas C (1997). Karyotypes of the most primitive catfishes (Teleostei: Siluriformes: Diplomystidae). Journal of Zoological Systematics and Evolutionary Research.

[ref-15] Campos H, Dazarola G, Dyer BS, Fuentes L, Gavilán JF, Huaquín L, Martínez G, Meléndez R, Pequeño G, Ponce F, Ruiz VH, Sielfeld W, Soto D, Vega R, Vila I (1998). Categorías de conservación de peces nativos de aguas continentales de Chile. Boletín Del Museo Nacional De Historia Natural.

[ref-16] Centro de Ecología Aplicada (2008). Estudio de impacto ambiental Proyecto Hidroeléctrico Aysén. Línea de base, Cap. 4, Medio biótico—Flora y fauna acuática. p 987. https://www.e-seia.cl/archivos/20080812.113038.pdf.

[ref-17] Cifuentes R, González J, Montoya G, Jara A, Ortíz N, Piedra P, Habit E (2012). Relación longitud-peso y factor de condición de los peces nativos del río San Pedro (cuenca del río Valdivia, Chile). Gayana (Concepción).

[ref-18] Colin N, Piedra P, Habit E (2012). Variaciones espaciales y temporales de las comunidades ribereñas de peces en un sistema fluvial no intervenido: Río San Pedro, Cuenca del Río Valdivia (Chile). Gayana (Concepción).

[ref-19] Curoto L (2015). Contribución molecular a la biogeografía del género *Diplomystes* (Teleostei: Siluriformes) en Sudamérica. Thesis Licenciatura en Biología.

[ref-20] Cussac VE, Habit E, Ciancio J, Battini MA, Riva Rossi C, Barriga JP, Baigún C, Crichigno S (2016). Freshwater fishes of Patagonia: conservation and fisheries. Journal of Fish Biology.

[ref-21] Dingerkus G, Uhler LD (1977). Enzyme clearing of alcian blue stained whole small vertebrates for demonstration of cartilage. Stain Technology.

[ref-22] Dray S, Dufour AB (2007). The ade4 package: implementing the duality diagram for ecologists. Journal of Statistical Software.

[ref-23] Duarte W, Feito R, Jara C, Carlos M, Orellana AE (1971). Ictiofauna del sistema hidrográfico del río Maipo. Boletín Del Museo Nacional De Historia Natural.

[ref-24] Duméril AMC (1856). Ichthyologie analytique ou essai d’une classification naturelle des Poissons, à l’aide de tableaux synoptyques.

[ref-25] Dyer BSD (2000). Systematic review and biogeography of the freshwater fishes of Chile. Estudios Oceanológicos.

[ref-26] Eigenmann CH (1890). The evolution of catfishes. Zoe.

[ref-27] Eigenmann CH (1927). The fresh-water fishes of Chile. Memories of the National Academy of Sciences.

[ref-28] Ferraris CJ, Reis RE, Kullander S, Ferraris CJ (2003). Family Diplomystidae (Velvet catfishes). *Checklist of freshwater fishes of South and Central America*.

[ref-29] Ferraris CJ (2007). Checklist of catfishes, recent and fossil (Osteichthyes: Siluriformes), and catalogue of siluriform primary types. Zootaxa.

[ref-30] Fink SV, Fink WL (1981). Interrelationships of the ostariophysan fishes (Teleostei). Zoological Journal of the Linnean Society.

[ref-31] Fink SV, Fink WL, Stiassny MIJ, Parenti L, Johnson GD (1996). Interrelationships of the ostariophysan fishes. *Interrelationships of fishes*.

[ref-32] Girard C, Gilliss JM (1855). Appendix F. Fishes. The United States naval astronomical expedition to the Southern Hemisphere, during the years 1849-’50-’51-’52.

[ref-33] Grande L (1987). Redescription of *Hypsidoris farsonensis* (Teleostei: Siluriformes), with a reassessment of its phylogenetic relationships. Journal of Vertebrate Paleontology.

[ref-34] Habit E (1994). Ictiofauna en canales de riego de la cuenca del rio Itata durante la época de Otoño-Invierno. Boletín De La Sociedad De Biología De Concepción.

[ref-35] Habit E (2005). Aspectos de la biología y hábitat de un pez endémico de Chile en peligro de extinción (*Diplomystes nahuelbutaensis* Arratia, 1987). Interciencia.

[ref-36] Habit E, Jara A, Colin N, Oyanedel A, Victoriano P, Gonzalez J, Solis-Lufí K (2009). Threatened fishes of the world: *Diplomystes camposensis*. Environmental Biology of Fishes.

[ref-37] La Cépéde BG (1803). Histoire naturelle des poissons.

[ref-38] Legendre P, Legendre L (2012). Numerical ecology.

[ref-39] Leybold F (1859). Descripción de una nueva especie de pez, descubierto por don Federico Leybold en el Río Seco de los baños de Colina. Anales De La Universidad De Chile.

[ref-40] López HL, Menni RC, Donato M, Miquelarena AM (2008). Biogeographical revision of Argentina (Andean and Neotropical Regions): an analysis using freshwater fishes. Journal of Biogeography.

[ref-41] Lundberg JG, Baskin JN (1969). The caudal skeleton of catfishes, order Siluriformes. American Museum Novitates.

[ref-42] Lundberg JG, Berra TM, Friel JM (2004). First description of small juveniles of the primitive catfish *Diplomystes* (Siluriformes, Diplomystidae). Ichthyological Exploration of Freshwaters.

[ref-43] Mabee PM, Grey EA, Arratia G, Bogutskaya N, Boron A, Coburn MM, Conway KW, He S, Naseka A, Rios N, Simons A, Szlachciak J, Wang X (2011). Gill arch and hyoid arch diversity and cypriniform phylogeny: distributed integration of morphology and web-based tools. Zootaxa.

[ref-44] Mac Donagh EJ (1931). Notas zoológicas de una excursión entre Patagonia y San Blas. Notas Preliminares Del Museo De La Plata.

[ref-45] MINSEGPRES (2008). *Aprueba Y oficializa nómina para el tercer proceso de clasificación de especies según su estado de conservación*.

[ref-46] Molina JI (1782). Sagio sulla storia naturale del Chile.

[ref-47] Muñoz-Ramírez CP, Unmack PJ, Habit E, Johnson JB, Cussac VE, Victoriano P (2014). Phylogeography of the ancient catfish family Diplomystidae: biogeographic, systematic, and conservation implications. Molecular Phylogenetics and Evolution.

[ref-48] Muñoz-Ramírez CP, Victoriano P, Habit E (2015). Inter-basin dispersal through irrigation canals explains low genetic structure in *Diplomystes cf. chilensis*, an endangered freshwater catfish from Central Chile. Limnologica.

[ref-49] Philippi R (1866). *Bemerkungen über die chilienischen flussfische*.

[ref-50] Pinna MCCD, Malabarba LR, Reis RE, Vari RP, Lucena ZM, Lucena CAS (1998). Phylogenetic relationships of Neotropical siluriforms (Teleostei: Ostariophysi): historical overview and synthesis of hypotheses. *Phylogeny and classification of neotropical fishes*.

[ref-51] R Core Team (2016). https://www.R-project.org/.

[ref-52] Sabaj Perez MH (2014). http://www.asih.org/.

[ref-53] Sullivan JP, Lundberg JG, Hardman M (2006). A phylogenetic analysis of the major groups of catfishes (Teleostei: Siluriformes) using rag1 and rag2 nuclear gene sequences. Molecular Phylogenetics and Evolution.

[ref-54] Venables WN, Ripley BD (2002). *Modern applied statistics with S*.

[ref-55] Vila I, Contreras M, Fuentes L (1996). Reproducción de *Diplomystes nahuelbutaensis* Arratia, 1987 (Pisces: Diplomystidae). Gayana Oceanologica.

[ref-56] Zama A, Cárdenas E (1984). Descriptive catalogue of marine and freshwater fishes from the Aysén Region, southern Chile, with zoogeographical notes on the fish fauna. Introduction Iinto Aysén Chile of Pacific Salmon, no. 9.

